# The complex, graded vocal repertoire of the social Rüppell’s vulture

**DOI:** 10.1371/journal.pone.0360246

**Published:** 2026-10-21

**Authors:** Connor Proudfoot, Charissa N. Brons, Maarten W. A. Vis, Anne Marijke Schel, Eva S. J. van Dijk, Jorg J. M. Massen

**Affiliations:** 1 Animal Behaviour and Cognition, Utrecht University, Utrecht, The Netherlands; 2 Vulture Cognition Lab, Utrecht University, Utrecht, The Netherlands; 3 Diergaarde Blijdorp, Rotterdam, The Netherlands; 4 Vogelpark Avifauna, Alphen aan den Rijn, The Netherlands; Texas Christian University, UNITED STATES OF AMERICA

## Abstract

The evolutionary origins of complex communication remain contentious, with social, ecological, and environmental factors proposed as selective drivers. The Social Complexity Hypothesis for Communicative Complexity (SCHCC) predicts that the cognitive demands of navigating socially intricate groups favour the evolution of complex communication. Although several studies provide support for the SCHCC, its empirical basis remains limited and inconsistent, as evidence is drawn predominantly from a small number of well-studied taxa (primarily primates), often neglects species’ complete vocal repertoires, and employs inconsistent approaches for assessing social and vocal complexity. This narrow focus limits its generalisability across animals, underscoring the need to characterise the full communicative complexity of additional model species to expand its evolutionary relevance. Vultures, with their varied social structures, provide a unique system to test this hypothesis, yet foundational work on communication in this guild is limited. Here, we present the first quantitative analysis of the vocal repertoire of the critically endangered, highly social Rüppell’s vulture, based on 2,500 calls recorded from two captive populations (35 individuals) across diverse social contexts. Using a fuzzy clustering approach, we reveal a repertoire far more complex than previously assumed, comprising multiple discrete and graded call types, with gradations likely conveying additional information. Calls serve diverse functions, including mediating parent-offspring interactions, manipulating conspecifics, and resolving conflicts. The Rüppell’s vulture repertoire exceeds the average call repertoire size observed across birds and is likely an adaptation to this species’ social life. Our results further establish vultures as a promising model for studying the vocal evolution and provide a framework for comparative analyses that can illuminate the selective pressures shaping complex communication.

## Introduction

Primates, particularly humans, represent an extreme case of encephalisation and intelligence. One leading hypothesis, the social brain hypothesis, suggests their extreme social complexity drove their exceptional cognitive evolution [1–5]. Although this hypothesis has received some support [4,6–15], empirical evidence remains mixed with alternate pressures such as ecology and environment also influential [16–24]. Moreover, differences in neural architecture further complicate its application beyond mammals, particularly in birds, where neuronal density is often a stronger predictor of cognitive performance than brain size [25], and in insects, which achieve remarkable behavioural complexity despite having relatively few neurons [26]. Consequently, there is growing recognition that broad hypotheses linking sociality to general intelligence should be complemented by tests of specific social-cognitive domains. Communication represents one such domain, being inherently social, giving rise to the Social Complexity Hypothesis for Communication Complexity (SCHCC), which predicts that increasingly complex social systems favour increasingly complex communication [3,27,28].

Animal signals span diverse sensory modalities and carry direct fitness consequences, with sound signals being particularly prominent among vertebrates. The ability to orchestrate sounds for communication evolved independently across diverse phylogenetic lineages and is the most researched signalling modality [29–35] Despite its widespread emergence, however, species vary strikingly in the complexity of their acoustic signalling systems through, for example, repertoire size, call gradation and flexibility, and sound sequences and combinations [36,37]. As such, explaining how and why this diversity evolved remains a major goal of evolutionary biology, particularly to illuminate the possible pathways shaping the evolution of human language [5,38].

In line with the SCHCC, complex vocal communication is regularly found in animals living in complex societies, where individuals regularly interact and where managing cooperation and competition are important for individual fitness [2,39]. In support of this hypothesis, researchers have identified complex communication in many socially complex species (e.g., chimpanzees (*Pan troglodytes*) [40], African elephants (*Loxodonta africana*) [32,41], bottlenose dolphins (*Tursiops truncatus*) [42], Parid songbirds [43]), and cooperatively breeding species (e.g., meerkats (*Suricata suricatta*) [44], grey wolves (*Canis lupus*) [45], and birds across diverse lineages [46,47]). Further comparative evidence stems from non-human primates, as a taxonomic group, with studies linking social complexity to complexity of a species’ communication systems [48,49]. Additionally, social living in rodents has been linked to both expanded vocal repertoires and the emergence of specific calls that buffer the costs of increased interaction rates [50], while in bats and swallows, call information content likewise increases with species-level social complexity [51,52]. Therefore, because signals operate within a social context, evolutionary investment in signal production, perception, and interpretation is expected only when the cost of not communicating outweighs the energetic burden of maintaining these cognitive abilities [27].

Nevertheless, despite the evidence presented, the evolutionary processes driving communicative complexity remain poorly resolved. Empirical support for the SCHCC is inconsistent both within and beyond traditional model taxa [46,53–57], with different studies identifying different facets of sociality as primary drivers of communicative complexity and no consistent pattern emerging across taxa. In birds, for example, cooperative breeding has been associated with larger functional repertoires, but evidence for broader effects of sociality is less clear [46,47]. Their analysis of social cohesiveness detected a significant difference in repertoire size only between species with minimal social cohesiveness (no persistent social bonds) and those with high social cohesiveness (maintaining stable, year-round social bonds), whereas moderately cohesive species were statistically indistinguishable from both, highlighting an ambiguous pattern for intermediate social forms. Their results therefore support an association between the extremes of social cohesion and communicative complexity, but do not demonstrate a simple continuous relationship between increasing social cohesiveness and repertoire size. In contrast, another study in birds found no association between sociality (mating system) and vocal complexity [53]. At the same time, ecological and environmental factors influence communication, while phylogenetic history may constrain its divergence, further complicating interpretations of vocal evolution and expression [28,47,58–61]. Several factors therefore currently limit broad comparative conclusions, and expanding the taxonomic breadth of study systems is essential for evaluating the generality of the SCHCC across the animal kingdom, alongside efforts to standardise measures of both sociality and communicative complexity [28].

Measuring acoustic complexity remains a major challenge in this area of research because the complexity of a vocal repertoire is determined not only by the number of calls an animal produces but also by the overall structure and use of its vocal repertoire [36,62]. Repertoires can be fully or partially discrete or graded, depending on acoustic variation within and between call types. Discrete repertoires are characterised by clearly defined call types with minimal within-call variation and no acoustic overlap [36,62]. In contrast, graded repertoires feature overlapping call structures and substantial within-call variation, enabling more flexible and informative signalling. Such within-call type variation may function to, for example, signal arousal (e.g.[63,64],), individual identity (e.g., [65–67]), or both (e.g.[64,68],). Call variation may also result from physiological changes associated with shifts in emotional state, which influence respiratory intensity and vocal motor control, thereby shaping the acoustic structure of the produced signal; notably, even discrete calls may exhibit within-call variation that conveys additional information. Many species express a mix of both discrete and graded calls within their repertoire [36,62].

While the pressures selecting for increased vocal gradation remain under debate, ecological, environmental and social factors have, again, been implicated. Greater vocal gradation is often associated with open habitats, elevated predation risk, and complex social systems [62,69,70]. In macaques, for instance, more tolerant species have greater structural flexibility in calls associated with agonistic encounters than despotic species, which is presumed to be an adaptation to the greater dominance uncertainty associated with less steep and less stable rank orders [70]. However, recent evidence challenges this view, showing that Etruscan shrews (*Suncus etruscus*), which live in closed habitats and relatively simple social structures, have also evolved vocal gradation, albeit within a simple vocal repertoire [58]. In addition to gradation, call combinations add another layer of complexity to many acoustic signalling systems, in which simpler sound units are sequenced to modify the meaning of a call or convey additional information to receivers (reviewed in [71,72]). In some cases, these sequences are governed by syntactic rules that constrain how they are constructed [73,74]. Therefore, vocal complexity arises from the diversity of acoustic signals used, the gradation in their production, and in how multiple calls are combined sequentially; as such, studies testing the drivers of communicative complexity should consider all these properties.

Testing the generality of the SCHCC requires expanding comparative analyses beyond traditional primate models to underexplored and distantly related taxa, such as birds, using full vocal repertoires to better evaluate how social complexity shapes communicative evolution and its broader evolutionary convergence. For this, vultures represent a particularly valuable yet largely overlooked model [75,76]. At first glance, scavenging birds may seem unlikely candidates for studying the evolution of communication, having been historically portrayed as largely silent and solitary, but they are promising for several reasons. First, even when accounting for their large bodies, vultures have disproportionately large brains [75,77], which may support complex behaviours and cognition. Their large brains are likely, at least in part, an adaptation for locating two unpredictable resources essential to their survival, namely carcasses for food and thermal updrafts for low-cost movement [78]. A considerable amount of work has already shown that vultures use visual and social, as well as acoustic information to inform flight decisions, improving access to these ephemeral resources [78–81]. Notably, their successful responses to playback experiments using natural predator–prey sound stimuli suggest that vultures have reasonably good hearing [80]. Additionally, foraging *Gyps* vultures attend to neighbours’ flight patterns which increases their probability of finding a meal, however this also means that, once a carcass is discovered, dozens of vultures often assemble within minutes. Although social foraging may reduce the energetic costs of locating food, it inevitably results in larger feeding aggregation at carcasses, where vultures gorge rapidly to fill their distensible crops, leading to exceptionally intense competition over the food resource, known as trophic competition. As more individuals arrive, both access to food and the rate at which it can be consumed decline sharply [82,83]. Under the SCHCC, this high-stakes, competitive feeding environment should select for the evolution of communicative strategies that minimise conflict and maximise food intake.

Moreover, all 23 vulture species, spanning three paraphyletic clades, are at least somewhat social and interact with conspecifics throughout their lives. Among Accipitrid vultures, long-term social monogamy and biparental care are common and alone are likely to select for a baseline level of social competence [82,84]. Yet, there is extreme variation in the overall social complexity between species, with marked differences in social structure, social organisation, mating system, and care systems, the four pillars of social complexity [75,76,84]. Species like the lappet-faced vulture (*Torgos tracheliotos*) and white-headed vulture (*Trigonoceps occipitalis*) tend to be more solitary and territorial; they forage alone, defend breeding areas, and spend little time interacting with conspecifics, perhaps occasionally meeting when feeding. In contrast, species of the *Gyps* genus are highly social and feed primarily on large ungulate carcasses, with much of their behavioural ecology relying on social information and interaction; they breed in colonies, forage in loose groups, and often gather at carcasses where competition is high. Even within this social genus, species differ in their breeding behaviour. For example, white-backed vultures (*Gyps africanus*) nest loosely across scattered treetops, while griffon vultures (*Gyps fulvus*), Rüppell’s vultures (*Gyps rueppelli*), and Cape vultures (*Gyps coprotheres*) breed in massive cliff-face colonies that house hundreds or even thousands of closely packed breeding pairs [76,82]. Griffon vultures have also previously been shown to occupy multi-layered social networks, with such complex social constellations potentially fostering more complex and diverse social interactions [85], stressing the need for effective communication. This makes the *Gyps* vultures among the most socially complex birds of prey and an ideal model system to test the SCHCC.

Vultures are thus a valuable and important taxonomic group for studying the evolution of social cognition and communication, and a systematic evaluation of their communicative abilities is long overdue. Vultures, and Accipitriformes in general, have received limited attention regarding their acoustic communication. This is largely due to the assumption that their vocalisations are simple and unremarkable, especially when compared to the more extensively studied songs of passerines [86–89]. Rüppell’s vulture societies, like those of other *Gyps* vultures, have relatively complex structures, with consistent dominance hierarchy and differentiated social ties that go beyond the pair-bond [90]. This social complexity is particularly extreme when compared to other birds of prey and therefore may have been a driver for complex communication under the SCHCC. However, a chasm lies between the social complexity of Rüppell’s vultures and their purported communicative complexity, being widely characterised as silent, with a restricted repertoire of basic signals [86,87]. These preconceptions, however, conflict with recent findings in the griffon vulture, the sister species of the Rüppell’s vulture, in which Romani et al. [89] identified a surprisingly diverse acoustic system featuring at least 12 distinct calls associated with diverse social contexts. This placed griffon vulture vocal complexity among the larger call repertoire sizes in birds [47,89], marking a significant advancement in understanding of vulture communication.

The Rüppell’s vulture diverged from the griffon vulture during the early Pleistocene, less than two million years ago, and both species retain highly social lifestyles, being colonial breeders and social foragers [75,76,90–92]. Accordingly, a critical question arises as to whether Rüppell’s vultures demonstrate comparable acoustic richness. If their communicative complexity is shared, it may reflect an ancestral trait that evolved in a socially complex common ancestor and was preserved in both species, as predicted by the social complexity hypothesis for communication. Therefore, we hypothesise that Rüppell’s vultures also possess a rich and graded vocal repertoire, with calls flexibly used to navigate a range of social contexts.

However, before addressing ultimate questions about communication from an evolutionary perspective in new taxa, researchers must first establish a species’ vocal repertoire: an acoustic ethogram cataloguing all sound signals produced by individuals over their lifetime [36,89,93]. In addition, understanding the context of calls is essential for interpreting their function and is a crucial step [89,94,95]. Establishing a vocal repertoire, and the potential of gradation within it, is methodologically challenging due to the lack of a standardised framework rendering comparisons between species challenging and therefore require expert knowledge to replicate [93,96,97]. Studies in this area often use vastly different approaches, including dimension reduction and discrimination techniques (e.g.[89,94,98–100],), supervised or unsupervised hard clustering approaches (e.g.[67,101,102],), and, more recently, soft clustering algorithms (e.g.[36,58,62,70,103,104],). For comparative consistency, it is thus paramount to make an informative but consistent choice in analytics. Given the potential gradation within the *Gyps* vocal repertoire highlighted by Romani et al. [89], we therefore apply fuzzy set theory [105], an increasingly popular unsupervised clustering method for bioacoustics, to offer a novel perspective on vocal structure and quantitatively assess vocal gradation in *Gyps* vultures [36,62]. By characterising the structure and functions of the Rüppell’s vulture vocal repertoire, our study addresses a critical gap in their behavioural ecology and contributes to broader discussions on the evolution of complex communication systems.

## Methods

### Subjects and housing

Here, we studied two groups of socially housed Rüppell’s vulture at Vogelpark Avifauna, Alphen aan den Rijn (n = 23), and Diergaarde Blijdorp, Rotterdam (n = 8 adults, and 4 chicks), the Netherlands (S1 Table). The study population included both captive-bred and wild-caught Rüppell’s vultures (confiscated from the illegal wildlife trade in Italy, 2005), with a near-equal sex ratio across both locations, allowing the recording of chicks pre-hatching and of individuals up to at least 25 years old (the age of birds that were confiscated from illegal trade is unknown, but they were at least adults, based on their plumage in 2005). Alongside Rüppell’s vultures, other species in the aviaries included white-headed vultures (*Trigonoceps occipitalis*), white-backed vultures (*Gyps africanus*), hooded vultures (*Necrosyrtes monachus*), marabou storks (*Leptoptilos crumenifer*), black kites (*Milvus migrans*), pied crows (*Corvus albus*), southern ground hornbills (*Bucorvus leadbeateri*), and various smaller passerine and galliform species. Our study population comprised individuals in breeding pairs (n = 16), including one male-male couple, unpaired adults (n = 8), juveniles (n = 7), and chicks (n = 4). Individuals were identified by physical features and unique leg-rings (see Fig 1A, H, I). Feeding occurred at least three times a week in both zoos, aligning with their irregular natural foraging regime. Food consisted of diverse carrion depending on availability and water was available *ad libitum*. Housed in zoos, all vultures were well habituated to human observers prior to this study.

**Fig 1:**
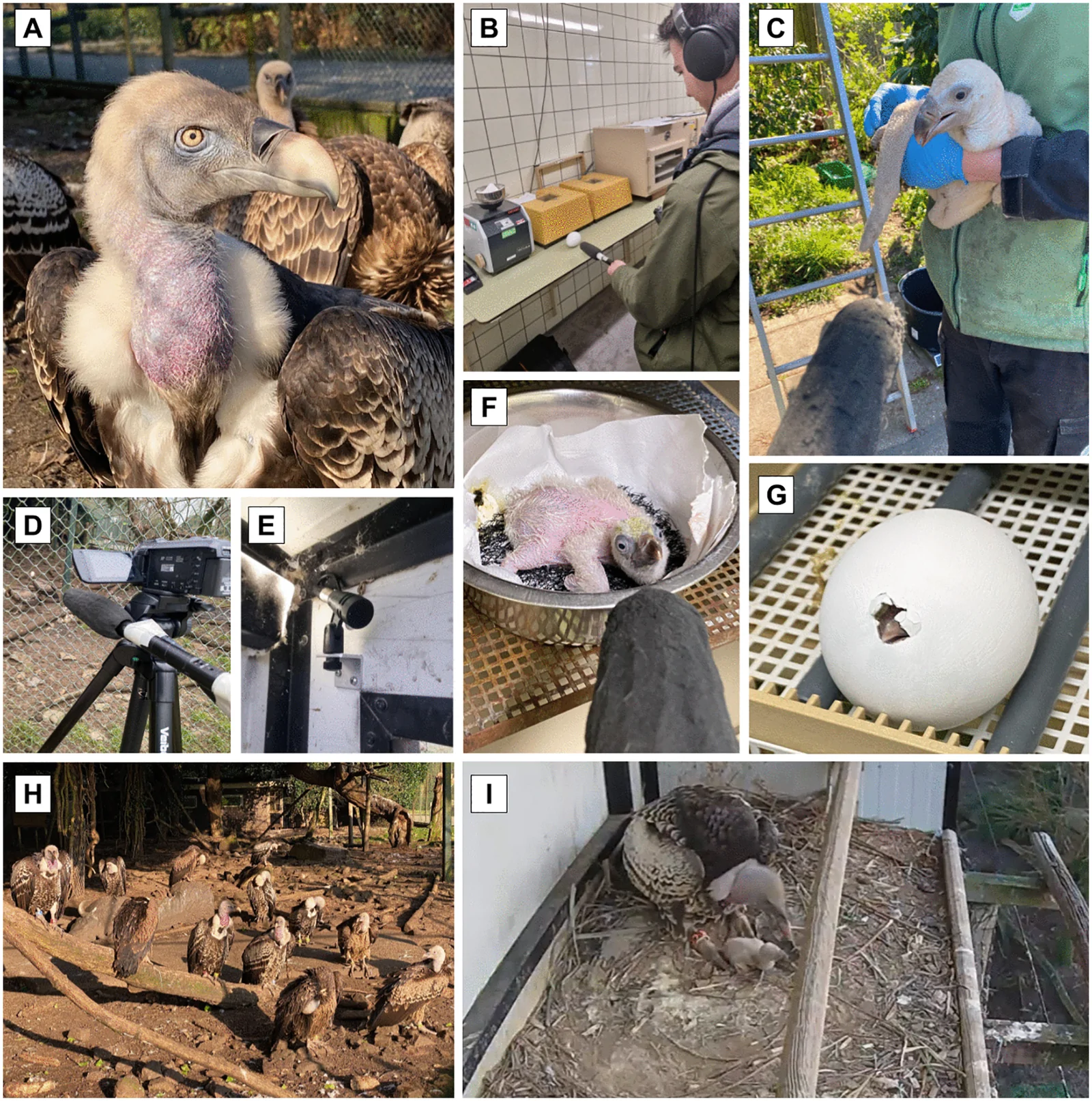
Overview of the data collection procedure for assessing the Rüppell’s vulture vocal repertoire across Vogelpark Avifauna and Diergaarde Blijdorp, conducted from September 2024 to May 2025. **(A)** Adult male Rüppell’s vulture, Stan, aged 25 + years. **(B)** Recordist C.P. sampling pre-hatching egg vocalisations from an internal pipped Rüppell’s vulture egg (25G4) in the incubation room at Diergaarde Blijdorp. **(C)** One-month-old chick 25G4 recorded during a veterinary procedure, including ringing and weighing, at Diergaarde Blijdorp. **(D)** Camera and microphone setup on a tripod used for recording during general group and feeding contexts. **(E)** Nest microphone installed for passive monitoring of parent–chick vocalisations in nest 3 at Diergaarde Blijdorp. **(F)** Chick 25G15 one day after hatching, being recorded in the incubation room at Diergaarde Blijdorp. **(G)** Chick 25G15 breaking through the shell of its egg (external pip) while vocalising, recorded in the incubation room at Diergaarde Blijdorp. **(H)** Group of Rüppell’s vultures resting in the sun at Vogelpark Avifauna. **(I)** View from the nest camera in nest 3 at Diergaarde Blijdorp, showing Ayla (mother) feeding chick 25G4. Photos: Connor Proudfoot.

### Sampling procedures

We conducted observations from 27 September 2024–31 May 2025, using ad libitum sampling [106] with various recording protocols to capture as many vocalisations as possible across diverse contexts. These dates span the non-breeding (September to November), mating (from December to February) and chick-rearing (March to August) periods in these captive colonies, and thus almost the full spectrum of social contexts. First and foremost, we recorded 10-minute general group sessions at Vogelpark Avifauna, focused on covering diverse social interactions. During recording, a microphone and camera were directed towards areas of the aviary with the highest vulture activity or directed at specific dyads or groups engaged in social interactions (Fig 1H). The caller and the apparent receiver, along with the behaviours occurring concurrently, were spoken into the microphone. Receivers were identified as the individual to whom the caller directed its vocalisation, either in direct response to their behaviour or, in the case of threats, when the caller physically directed its beak and emitted sound towards them. We selected this ad libitum approach to maximise the number of calls collected and catalogue the structure of their vocal repertoire. Second, we recorded 30-minute feeding sessions in both groups. These sessions commenced when the first individual approached a new food source placed in the aviary by the caretakers, which often triggered a rapid influx of other vultures producing a cacophony of vocalisations under intense trophic competition (S1 Video in S1 File). In both contexts, the caller was always within 10 metres from the microphone.

Third, to capture sexual and parent-chick vocalisations, nest recordings were made using nest recording equipment placed in each of the three nest boxes occupied by Rüppell’s vulture pairs at Diergaarde Blijdorp (Fig 1E and 1I). These nest microphones were installed prior to the breeding season and fitted with a 5-metre cable to allow us to record the nest without disturbing the chick or parents. These recordings varied in time and were the longest in duration (median = 60 minutes) to avoid disturbing nesting and parental behaviour. Additionally, during the breeding season, vultures lay a single egg per clutch, which is removed and artificially incubated at Diergaarde Blijdorp’s facilities, while the pairs are provided with a model egg. After hatching, the chicks are hand-reared for the first 3–7 days to enhance survival at this vulnerable age, before being fit enough to return to a nest to be reared by their (foster) parents. This procedure is employed to improve captive breeding success and is often combined with cross-fostering and double clutching [107]. This provided an opportunity to record both prenatal vocalisations (Fig 1B and 1G) and the calls of the semi-altricial chicks during hand-feeding sessions (Fig 1F). In the incubator, vocalisations were emitted at a high rate, allowing recording times to be minimised during this critical period. Upon return to the nest, we also used the nest microphones to record parent-chick interactions. Chicks were also opportunistically recorded during ringing at one month old (Fig 1C).

In addition to the regular recording protocols, we recorded during special events that provided unique contexts not encountered in the vultures’ daily routines. To supplement the standard feeding sessions, we collected recordings during another study which involved introducing a stuffed model spotted hyena (*Crocuta crocuta*) as a mammalian competitor during a feeding session at Vogelpark Avifauna on 27 March 2025, following two days of fasting to encourage context-specific vocalisations such as alarm calls. A similar procedure was conducted with a model snake (resembling a python and 160 cm long) placed next to their regular food on 3 April 2025, simulating a potential threat to the colony. Furthermore, since the vultures were housed with other large raptors, we could monitor their response to species which may pose a threat to their chicks (e.g., pied crows and black kites). Lastly, we opportunistically recorded during the capture of the entire group at Vogelpark Avifauna (15 April 2025), done for husbandry reasons, to document potential stress-related vocalisations during handling and veterinary procedures, as previously reported in griffon vultures (Romani et al., 2022). These procedures were part of different studies and were not considered animal experimentation, given ethical approval by Utrecht University’s animal welfare committee (IvD: WP number: 10863-2025-02).

Various bioacoustics tools were used throughout data collection. Initially, we recorded sessions with a Marantz PMD 660 solid-state recorder (until 10 January 2025), and from 14 January 2025 onwards we used a ZOOM H4 Essential solid-state recorder. For recording general group and feeding sessions, the recorder was connected to a Sennheiser MKE 600 shotgun microphone mounted on a tripod (Fig 1D). Recordings were made at a sampling frequency of 44.1 kHz, yielding a maximum frequency response of 22 kHz, and an audio bit depth of 16. Additionally, a Panasonic HC-V785 camcorder was used to capture the behavioural context associated with vocalisations. These recordings were made at approximately two to 10 m of the calling individual. The same recorder and microphone setup was used to record egg and chick vocalisations within an artificial incubator. For nest recordings, three Sennheiser E604 Dynamic instrument microphones were placed inside the nests, and sessions were video recorded using the internal camera system, which was paired with audio files to annotate behavioural contexts. All audio (.WAV format) and video (.MTS format) files were stored on a solid-state drive for later analysis.

### Acoustic and behaviour analysis

We labelled 2500 calls spanning a range of social contexts (i.e., non-agonistic, aggression, parental, sexual, and stress) (see Table 1 for detailed account). This sample size was predetermined and deemed sufficient to reliably represent the vocal repertoire of Rüppell’s vultures, while also reducing the computational demands of the analysis. For comparison, Wadewitz et al. [36] included 912 chacma baboon (*Papio ursinus*) vocalisations in a seminal bioacoustics fuzzy clustering analysis, while Romani et al. [89] characterised the griffon vulture vocal repertoire using 542 calls. A single, experienced observer (C.P.) performed bioacoustic analysis on spectrograms generated using default settings in Raven Pro (version 1.6.5, available at https://www.ravensoundsoftware.com/) [108]. We classified calls as distinct if there was a minimum interval of 0.2 seconds with no emitted sound energy, deemed sufficient to separate independent calls while binding multisyllabic calls (including grouping sequences of different calls with short gaps) [103]. We further analysed only the calls that produced visibly clear spectrograms without interference from background noises, such as other animals or weather, allowing us to reliably annotate calls and extract the parameters for analysis. The observer labelled the audio spectrograms by placing selection boxes indicating the minimum and maximum call frequencies and the start and end of the vocalisation. We determined both intra-rater and inter-rater reliability of these labels using the intersection over union (IoU) method [109] across 125 calls (5% of total calls). This method calculated the proportion of overlapping area from the two paired selection boxes, and the reliability of these labels was high both within (IoU = 0.822) and between (IoU = 0.735) raters (comparing C.P. to C.N.B).

**Table 1:** Ethogram of behaviours used to assign a context to each call.

Behaviour	Description
Social Isolation	The caller is alone, positioned more than one wingspan from the nearest conspecific, and not engaged in any social interactions. Behaviour may include self-directed or non-social activities.
Non-aggressive social proximity	The caller is within one wingspan of another vulture without showing any agonistic behaviour. Affiliation behaviours such as contact sitting or allopreening may occur.
Receive approach	Either the caller approaches another vulture, or is approached, reducing distance from beyond to within one wingspan.
Protect threat (food)	A vocalisation directed toward another vulture while the caller is in possession of and guarding a food resource.
Protect threat (space)	A vocalisation directed toward another vulture while the caller is defending its nest site, roosting location or space.
Demand access	A vocalisation directed toward a conspecific that is in possession of, or guarding, a food resource.
Receive aggression (food)	The caller vocalises in response to aggression from another vulture during a conflict over food. This includes both contact and non-contact (e.g., threats) aggression
Receive aggression (space)	The caller vocalises in response to aggression from another vulture during a conflict unrelated to food, such as over nesting or roosting space.
Sexual behaviour	The caller is engaged in sexual activity, such as mounting or copulation.
Juvenile-Parent	A recently fledged juvenile vocalises toward its parent(s) within the first three months post-fledging.
Parent to chick	A parent vocalises toward its dependent chick before it fledges the nest.
Chick not feeding	A chick vocalises in the presence or absence of a parent and is not being fed at the time.
Chick feeding	A chick vocalises while being fed by a parent.
Egg	The caller vocalises from within the egg prior to hatching.
Stress	The caller vocalises in response to a non-social, high-stress or negative context, including exposure to predators, handling, or veterinary procedures.

Because acoustic measurements can be noisy, Wadewitz et al. [36] suggest using large sets of collinear acoustic features to produce more stable clustering solutions. Therefore, we extracted 37 temporal and spectral acoustic parameters, selected based on those Erb et al. [103] used in their fuzzy analysis of orangutan calls (S2 Table). The vocal repertoire of *Gyps* vultures contains both monosyllabic (comprised of one short sound type or note) and multisyllabic calls (comprised of a composite of multiple sound type, or notes), therefore we manually counted the number of notes as an additional parameter [89,110]. Additionally, each call was given knowledge-based call type labels based on visual and acoustic features. The initial call-type scheme followed the griffon vulture repertoire described in Romani et al. [89] and was subsequently refined and updated by C.P. during extensive observations of Rüppell’s vulture calls during data collection. Many call types previously described in griffon vultures were also identified in our dataset, including the hiss, bray, bray-hiss, grunt, groan, metallic groan, and small groan. The chicken coo, chicken groan, juvenile scream, and sharp scream were likewise documented in both species; however, we renamed these call types to chick call, chick begging call, juvenile begging call, and screech, respectively, to better reflect their acoustic properties and the behavioural contexts in which they are produced. We also described several new call types, including the sex groan, egg call, provision call, and whistle-hiss. For all analyses, the acoustic parameters were z-score standardised (mean = 0, standard deviation = 1) to ensure the measurement scales did not affect clustering results. We generated spectrograms for selected calls using the *warbleR* package [111].

After analysing each audio file, we classified the behavioural context of the call using the corresponding video footage (Table 1). The 15 broad behavioural contexts included three non-agonistic, five aggressive, five parental, one sexual, and one stress-related category. All videos were coded; however, in some cases (633 calls), the context could not be determined because the individual which produced the call was unclear (for example during intense feeding aggregations where many individuals are vocalising). Consequently, 1867 calls were used to describe the behavioural context.

### Statistical analysis

We carried out a fuzzy clustering analysis across all datapoints in combination with a uniform manifold approximation and projection (UMAP) to explore the structure of the Rüppell’s vulture vocal repertoire. All analyses were conducted in RStudio (version 4.5.1), with the raw data and code fully available in [112]. Fuzzy clustering algorithms, such as fuzzy k-means (FKM), are unsupervised machine learning methods designed to uncover patterns and structure in high-dimensional datasets [36,105]. Unlike hard clustering approaches that assign each data point to a single, exclusive cluster, fuzzy clustering allows data points to have partial membership across multiple clusters [105,113]. Each data point receives a membership score from 0 to 1 that reflects its degree of belongingness to each cluster, with the total across all clusters summing to 1. This soft clustering approach is particularly well-suited for analysing complex biological systems such as animal vocal repertoires, which often contain graded transitions between signal types. In such cases, hard clustering may obscure meaningful variation and mask the graded nature of vocal systems [36,58,70,103,104,114].

Fuzzy k-means clustering relies on two key parameters. The first, μ (the fuzzifier), determines the degree of cluster fuzziness. When μ = 1, no overlap between clusters is permitted, and the algorithm functions similarly to hard clustering methods, assigning each data point exclusively to one cluster. As μ increases, cluster boundaries become more diffuse, membership scores are distributed more evenly, and clusters begin to merge. At sufficiently high values of μ, membership scores approach equal values across clusters (approximately 1/k), reducing discrimination between clusters. The second parameter, k, specifies the number of clusters to be identified in the dataset. Average fuzzy silhouette values can be computed for each combination of k and μ, measuring how similar an object is to its own cluster compared to other clusters, with higher silhouette coefficients indicating better separation and cohesion among clusters. Following Wadewitz et al. [36], we evaluated clustering solutions across a range of μ values and numbers of clusters, selecting a solution that combined high silhouette values with stability across neighbouring μ values [62,104,114]. This approach allows us to identify the solutions with high within-cluster cohesion and strong between-cluster separation [113]. To implement this, we ran an iterative search using the full set of acoustic variables with the ‘fkm’ function from the ‘fclust’ R package [115], specifying a range of k from 2 to 17 (one more than the number of call types we identified) and increasing μ from 1.1 to 3.0 in intervals of 0.1. At μ > 1.5, clusters became overly fuzzy, with memberships approaching 1/k. Therefore, we examined silhouette scores for solutions between μ = 1.1 and μ = 1.5. Across this range, the nine-cluster solution consistently yielded high fuzzy silhouette values peaking at μ = 1.4 (S1 Fig), indicating a stable and well-separated clustering structure. Although the 17-cluster solution produced the highest silhouette value at μ = 1.4, this peak was not maintained across neighbouring μ values, where its silhouette values were consistently lower. We therefore considered this solution less stable than the nine-cluster solution. Additional fuzzy cluster solutions for the next best numbers of clusters, 8 and 10, are provided in S2 Fig and S3 Fig, with these clusters mainly differing in how the highly variable metallic groans are partitioned.

Fuzzy clustering outputs a membership matrix, where each data point (i.e., individual call) is assigned a membership coefficient for every cluster. These coefficients indicate the degree to which a call belongs to each cluster. To assess how clearly a call belongs to a specific cluster, we calculate the typicality coefficient (TC) by subtracting the second-highest membership value from the highest for each data point. This provides a measure of cluster assignment strength for each vocalisation [36,62,104,114]. By averaging typicality coefficients across all calls within a given call type, we can assess how typical that call type is overall. To classify call types as discrete, intermediate, or graded, we calculated typicality and atypicality thresholds by adding or subtracting half the mean absolute deviation from the mean typicality, respectively [36,62,104,114]. Call types with median typicality coefficients above the upper threshold were considered discrete, those below the lower threshold as graded, and those in between as intermediate.

To visualise the structure of the Rüppell’s vulture vocal repertoire, we used the UMAP non-linear dimension reduction technique [93]. UMAP is based on topological analysis with strengths in balancing both local and global patterns in the dataset and performs better for visualising the latent structure of acoustic data than more common dimension reduction techniques like principal component analysis [93]. In our analysis, we generated a single UMAP using the ‘umap’ function from the ‘uwot’ R package (nearest neighbours = 250, minimum distance = 0.1, number of components = 2) [116], and coloured the datapoints based on the knowledge-based call labels determined, along with the unsupervised maximum cluster memberships from fuzzy clustering. This approach provides a clear means of visualising vocal repertoire patterns and exploring potential overlaps in call structure. We also performed an exploratory analysis on the highly variable metallic groans by examining within- and between-cluster variation in each acoustic parameter to identify those that account for their structural variability.

## Results

### Overview of the Rüppell’s vulture vocal repertoire structure

Fuzzy clustering analysis revealed that the vocal repertoire of Rüppell’s vultures has a partially graded structure, comprising both discrete and graded call types (Fig 2). A summary of all acoustic parameters per call type is given in S3 Table. Visualisation using a uniform manifold approximation and projection reveals clear overlaps between the knowledge-based call labels assigned during sound analysis (Fig 2A) and those determined by the fuzzy clustering algorithm (Fig 2B; See also S4 Table). Some clusters corresponded to single, acoustically typical call types (e.g., the sex groans in Fig 2A cluster almost entirely into Cluster 7 in Fig 2B), while others encompassed overlapping vocalisations or subdivided broader types into more specific subtypes based on acoustic features (e.g., the metallic groans in Fig 2A fall across three broad Clusters 3, 4, and 6 in Fig 2B). Five call types had median typicality coefficients (TCs) exceeding the typicality threshold of 0.53, six were classified as atypical, with median TCs below 0.26. Five call types had median typicality values in between the two thresholds and are intermediately graded (S5 Table). Note, because the output produces continuous membership values that reflect relative similarity to cluster centroids, clusters should be interpreted as regions of acoustic similarity in a continuous feature space rather than discrete categories. Accordingly, within-call variation reflects the combined influence of all sources of acoustic variation present in the data, which may include but are not limited to individual differences, developmental stage, affective state, morphological constraints, and recording noise, although these sources cannot be disentangled within the present analytical framework.

**Fig 2:**
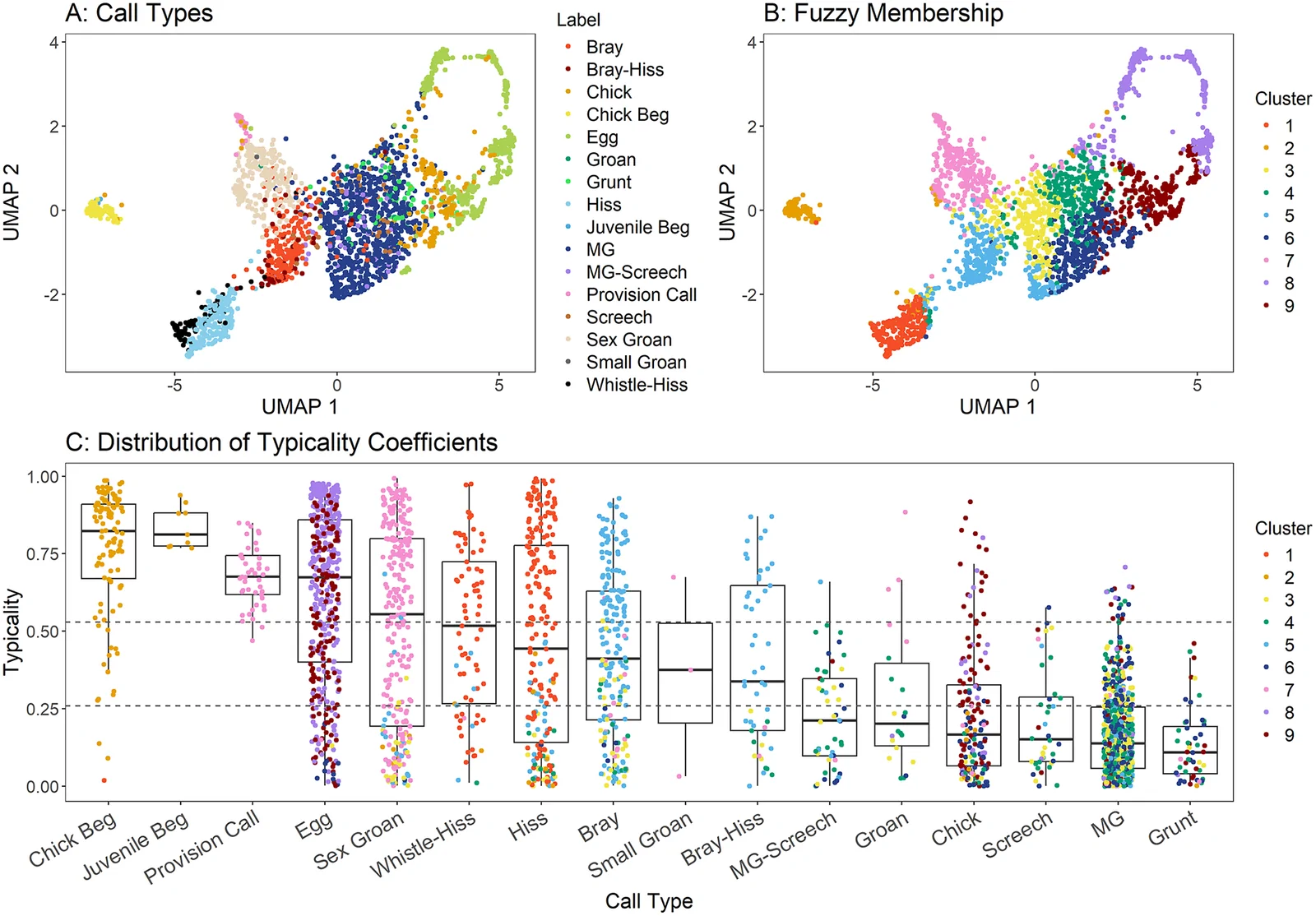
Visualisation of Rüppell’s vulture vocal repertoire structure. Two-dimensional UMAP projection of the structure of the Rüppell’s vulture vocal repertoire with calls coloured by (A) call type and (B) according to the cluster to which they show the highest membership, as determined by fuzzy k-means clustering. Plots use the same UMAP, meaning that individual datapoints on the same coordinates represent the same call in both. (C) Distribution of typicality coefficients across call types. Data points are coloured based on maximum cluster memberships corresponding to Fig 2B. The upper dashed line marks the typicality threshold, with calls above this line having a more discrete and prototypical structure. The lower dashed line indicates the atypicality threshold, below which calls are considered atypical and show a more graded structure. Box plots summarise the distribution of typicality values per call type, with thick horizontal lines representing the medians, boxes denoting the interquartile ranges (IQRs), and whiskers extending to the most extreme values within 1.5 times the IQR.

We also found a clear and strong association between call types and the contexts in which they are produced (Fig 3 and S6 Table). Most vocalisations occurred during conflicts, with distinct calls associated with receiving aggression, protecting a resource, or threatening an attack. Trophic competition was the context which elicited the greatest diversity of calls (nine calls in this context), of which five were unique to this context. Several call types were specific to young vultures, while one was produced exclusively during copulations. The small groan, a rare type of call, occurred only in non-aggressive social proximity. No unique vocalisations were recorded during feeding sessions involving hyenas or snakes, nor during veterinary procedures.

**Fig 3:**
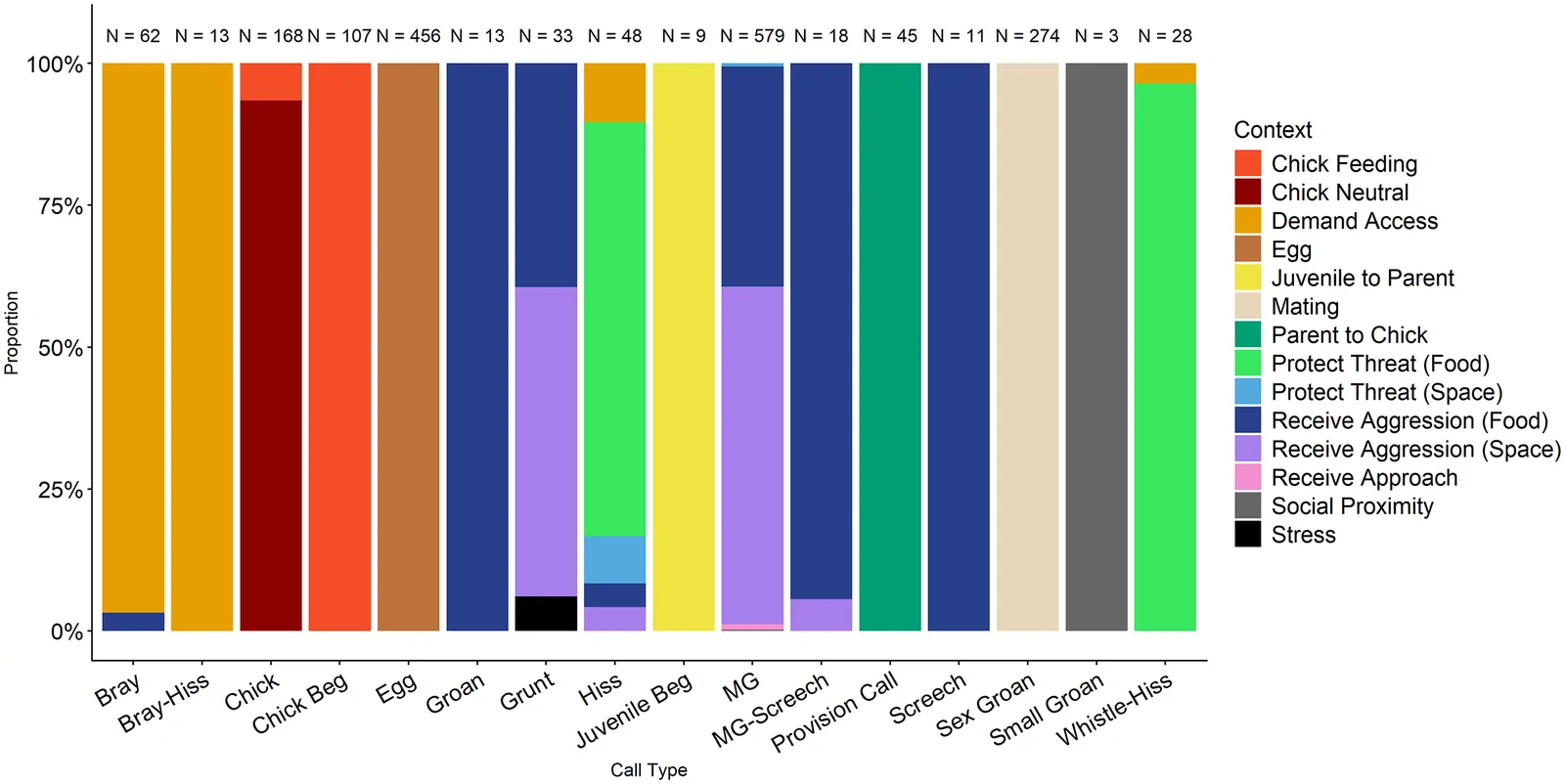
Rüppell’s vulture calls have a significant association with social contexts. Sample sizes per call are given above the stacked bar plots.

### Call descriptions and behavioural contexts

#### Egg, chick, and juvenile calls.

We identified four broad call types used by dependent young vultures, spanning from internal pipping to approximately three months post-fledging (Fig 4). The first vocalisations Rüppell’s vultures produce in their lifetime are soft, tonal “hmm” calls emitted from within the egg after the chick breaches the air chamber and begins pulmonary respiration (S1 Audio; S2 Video in S1 File). These internal pip vocalisations continue through hatching and seem to transition into post-hatching chick calls (referred to as chicken coos in [89]), characterised by similarly structured “hmm” calls that persist for several months (S2 Audio in S1 File). Given the husbandry procedure of artificial incubation, we could not determine whether parents also vocalise back to the chick in the egg in response to egg calls. Fuzzy clustering analysis revealed that these egg and chick calls overlap across Clusters 8 and 9, and acoustic differences between these call types likely result from whether the vocalisations are produced within the physical constraints of the eggshell or in open air and therefore forms the first major class of young vocalisations. Nevertheless, differences in the structural gradation of these two calls were striking, with egg calls being highly typical overall (median TC = 0.67, IQR = 0.46) however, after hatching, these vocalisations transitioned into more variable and inconsistent babble with lower typicality coefficients (median TC = 0.17, IQR = 0.26). Neither call was associated with any particular social context.

**Fig 4:**
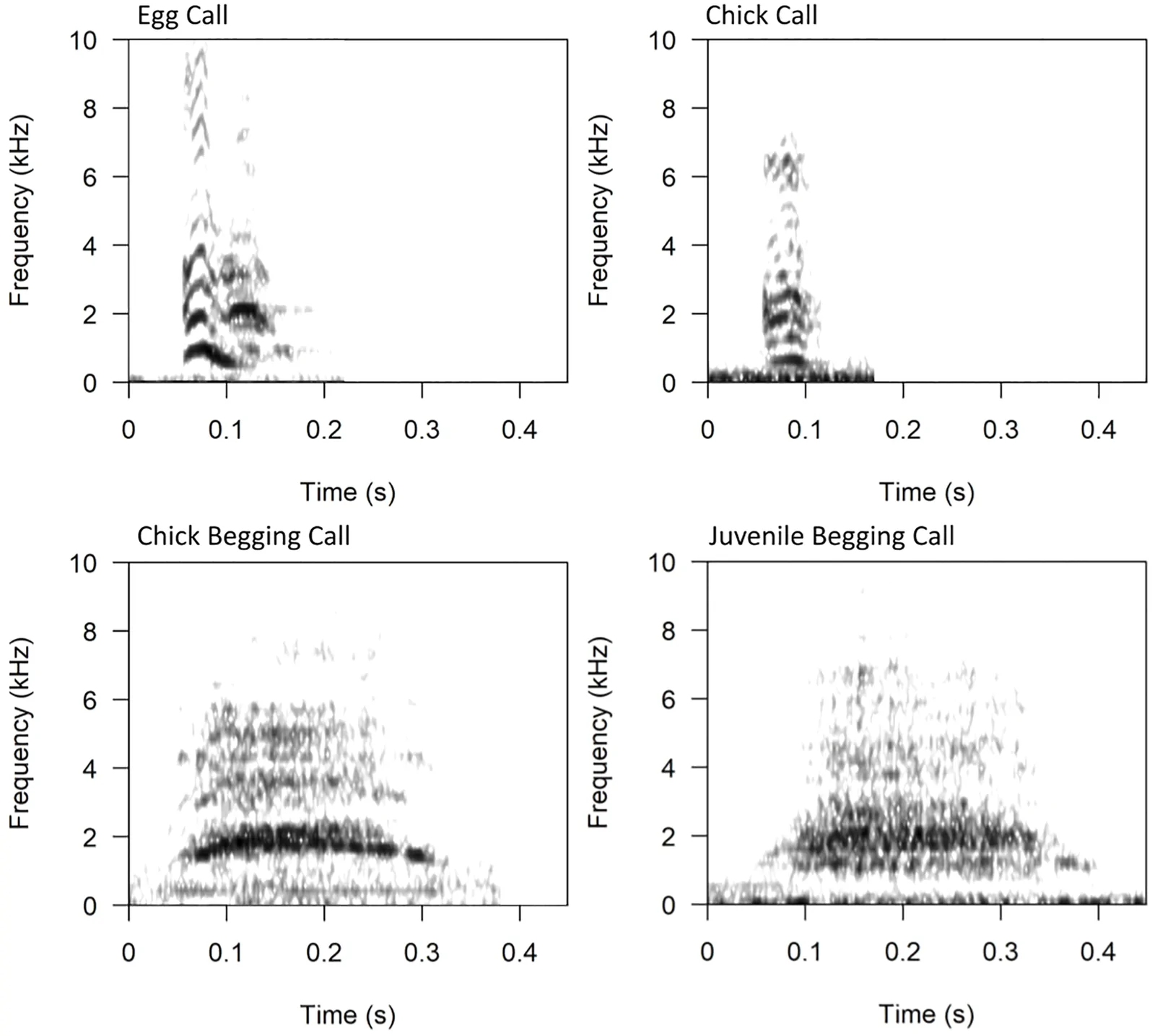
Vocalisations used by young Rüppell’s vultures when dependent on their parents for care.

The calls chicks and juveniles produce both in the nest and shortly after fledging, when begging for food from a parent, formed a highly distinct class (Cluster 2). Together, the chick begging calls (S3 Audio in S1 File; median TC = 0.82, IQR = 0.24) and juvenile begging calls (S4 Audio in S1 File; median TC = 0.81, IQR = 0.11) had the highest typicality of all calls and were highly distinct from all other calls (> 99% in Cluster 2). This difference was unlikely to be an artifact of recording protocols, as chick begs were recorded with nest microphones while juvenile begs were recorded with handheld equipment. Their similarity supports their combined classification as “begging screeches,” produced in the presence of a parent and likely functioning in food solicitation.

#### Provision call.

After returning to the nest after feeding, vulture parents regurgitate to feed their dependent chicks. During this time, we recorded the chick begging calls, while the parent would occasionally issue a guttural grunt while regurgitating which may signal to the chick that they are providing food (Fig 5; S5 Audio in S1 File). This call was not heard when individuals regurgitated food outside of the nest. This short, atonal call had a high typicality (median TC = 0.68, IQR = 0.13), and all of them clustered into Cluster 7.

**Fig 5:**
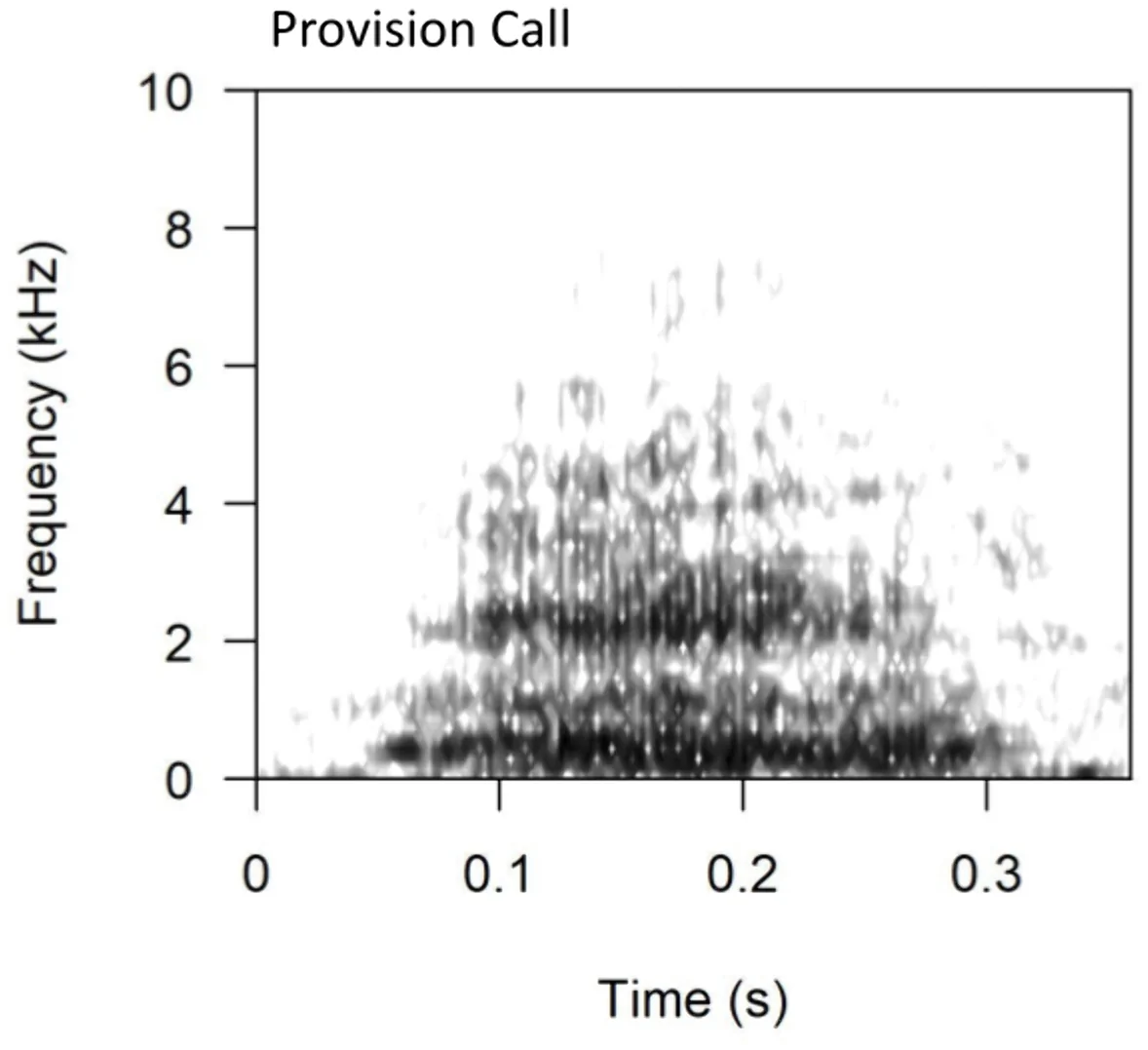
Provision call given by parent Rüppell’s vultures to their chicks when regurgitating food upon arrival in the nest after feeding.

#### Hisses and whistle-hisses.

Vultures hissed almost exclusively as threat calls (S6 Audio in S1 File), primarily when guarding meat from conspecifics (72.9%), and less commonly when defending nests or roosting space (8.3%) or demanding access to food (10.4%). This call was primarily given when individuals were competing for valuable resources and presented as a noise band on the spectrogram and were intermediately graded, with whistle-hisses (S7 Audio in S1 File) being more discrete than pure hisses (median TC: hiss = 0.44, IQR = 0.64; whistle-hiss = 0.52, IQR = 0.46). These calls differ, with hisses being a broad, atonal noise band, while whistle-hisses incorporate a more focused concentration of sound energy (Fig 6). Both vocalisations were often accompanied by ritualised threat displays, including wings spread, an elongated neck, foot stomping, and rapid jabbing neck movements directed at competitors. However, the factors influencing when vultures incorporate whistles into hisses, as opposed to producing pure hisses, remain unknown.

**Fig 6:**
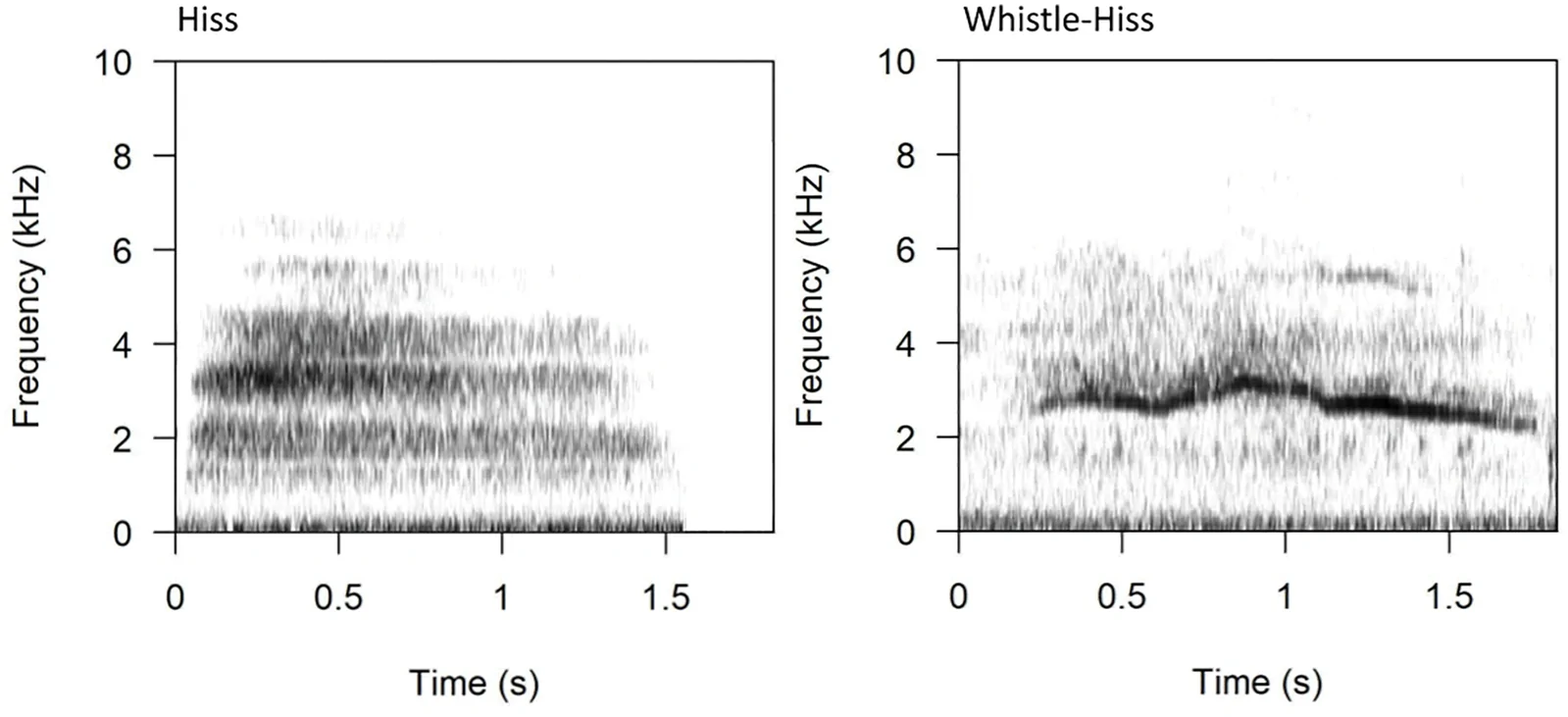
Spectrograms of hiss and whistle-hiss calls produced by Rüppell’s vultures. Hisses contain a broad, uniform band of noise energy, while whistle-hisses also consist of a noise band along with a narrower, more modulated concentration of acoustic energy.

#### *Brays and bray-hisses*.

Brays (S8 Audio in S1 File) and bray-hisses (S9 Audio in S1 File) were two call types associated exclusively with trophic competition, with both almost exclusively being emitted by an individual attempting to gain access to a guarded piece of food. Both form an intermediate call type with a moderately graded structure (median TC: bray = 0.41, IQR = 0.42; bray-hiss = 0.34, IQR = 0.47), which suggests some variability between calls. Qualitative observations suggest that brays vary depending on the receiver’s response, with individuals often repeating brays until they either gain access to the guarded meat or are actively threatened or chased away by the guard. In many cases, repeated braying was accompanied by an increase in intensity, particularly when the signal was ignored. As the interactions escalated toward a potential physical confrontation, the call often ended with an upward pitch inflection (Fig 7). This variation may have accounted for their moderately graded structure. Similarly, bray-hisses were more often incorporated when nearing direct attack, and may represent an intermediate call between the brays and hisses (Fig 7), with gradation in this overlap.

**Fig 7:**
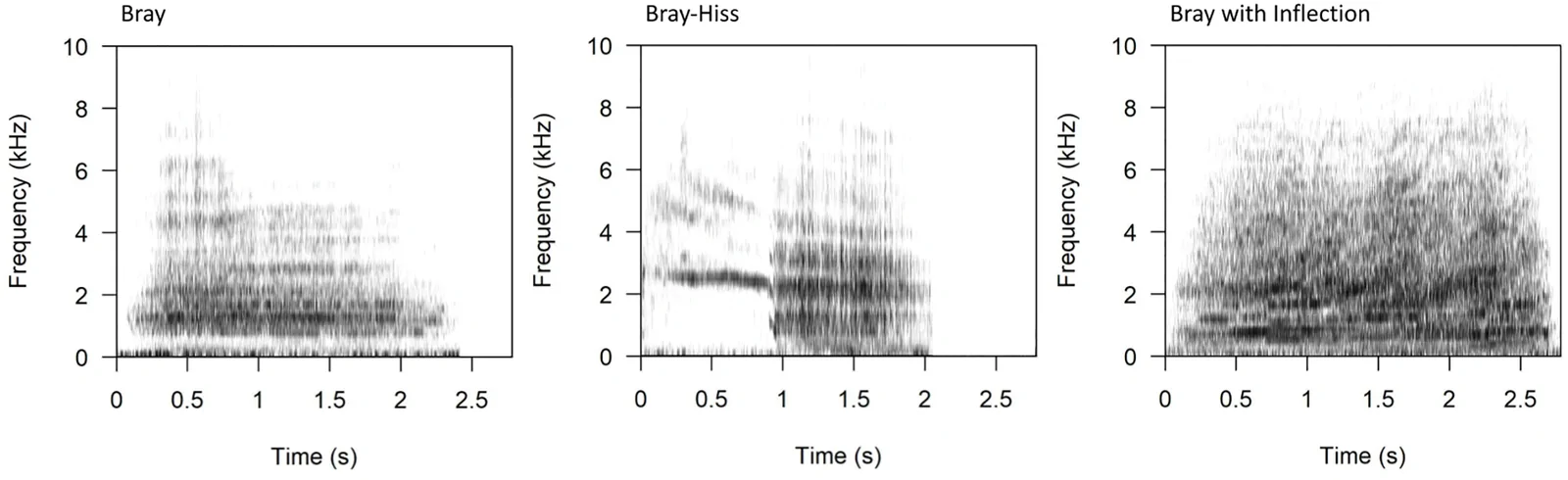
Spectrograms of Rüppell’s vulture bray and bray-hiss calls. The Bray-Hiss in the middle panel begins with a whistle-hiss element, which switches to a bray element at around 1 s. The rightmost panel shows a bray with an upwards frequency inflection.

#### Metallic groans.

Metallic groans accounted for one-third of all vocalisations recorded in this study and represented one of the most graded call type produced by Rüppell’s vultures (Fig 8; S10 Audio in S1 File). These calls form a broad vocal complex, predominantly associated with receiving aggression including physical attacks, threat displays, and non-contact aggression over food (38.9%) or space (59.4%). Metallic groans were also occasionally produced in seemingly non-agonistic encounters, such as when approached by a group member without visual signals of aggression (1.0%). The structure of metallic groans varied considerably in sequence length (see S11 Audio – S15 Audio in S1 File), ranging from single-note utterances to sequences of up to 43 notes (median = 5, IQR = 6). Due to this syntactic variation, 97.4% of the 727 metallic groans had typicality coefficients below the typicality threshold (median TC = 0.14, IQR = 0.20), indicating an extreme degree of acoustic variation and structural gradation (Fig 8). Furthermore, metallic groans did not cluster as a single unit but were distributed across all clusters except Cluster 1 which contained the broadband, atonal hisses and whistles. Nevertheless, 80.2% of metallic groans were concentrated within Clusters 3, 4, and 6.

**Fig 8:**
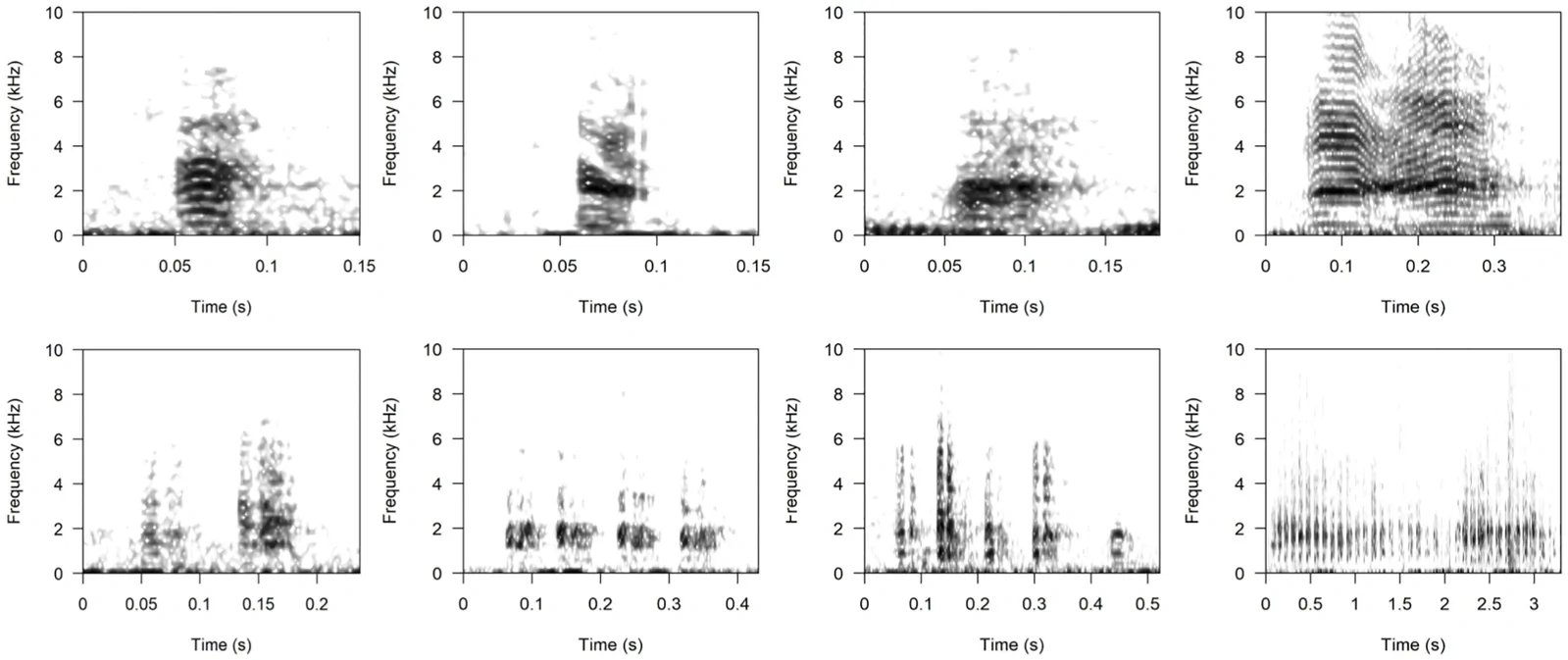
Structural and combinatorial diversity of metallic groans. The upper four spectrograms illustrate single-note metallic groans, each varying in their acoustic structure. The lower four spectrograms demonstrate how these individual notes are combined into longer, multi-note sequences, highlighting the flexible and graded nature of this call type. Note that the time scales differ between panels.

Given the extreme variability and graded nature of metallic groans, we quantified both between-cluster and mean within-cluster variation to identify acoustic parameters that differed across maximum fuzzy cluster assignments. Several parameters showed greater between- than within-cluster variation (S7 Table), with temporal features showing the strongest differences. Call duration and note number were of particular interest, as metallic groans consist of variable-length sequences of syllables, suggesting that these parameters underpin much of the gradation observed. To examine this, we compared cluster membership with these parameters [58]. As illustrated in Fig 9, both call duration and note count are reflected in the cluster assignments: Cluster 5 predominantly contains longer metallic groans with more notes, whereas Clusters 4 and 6 mainly include metallic groans composed of fewer than five notes. The extent to which these acoustic differences reflect variation in behavioural context, however, remains unclear.

**Fig 9:**
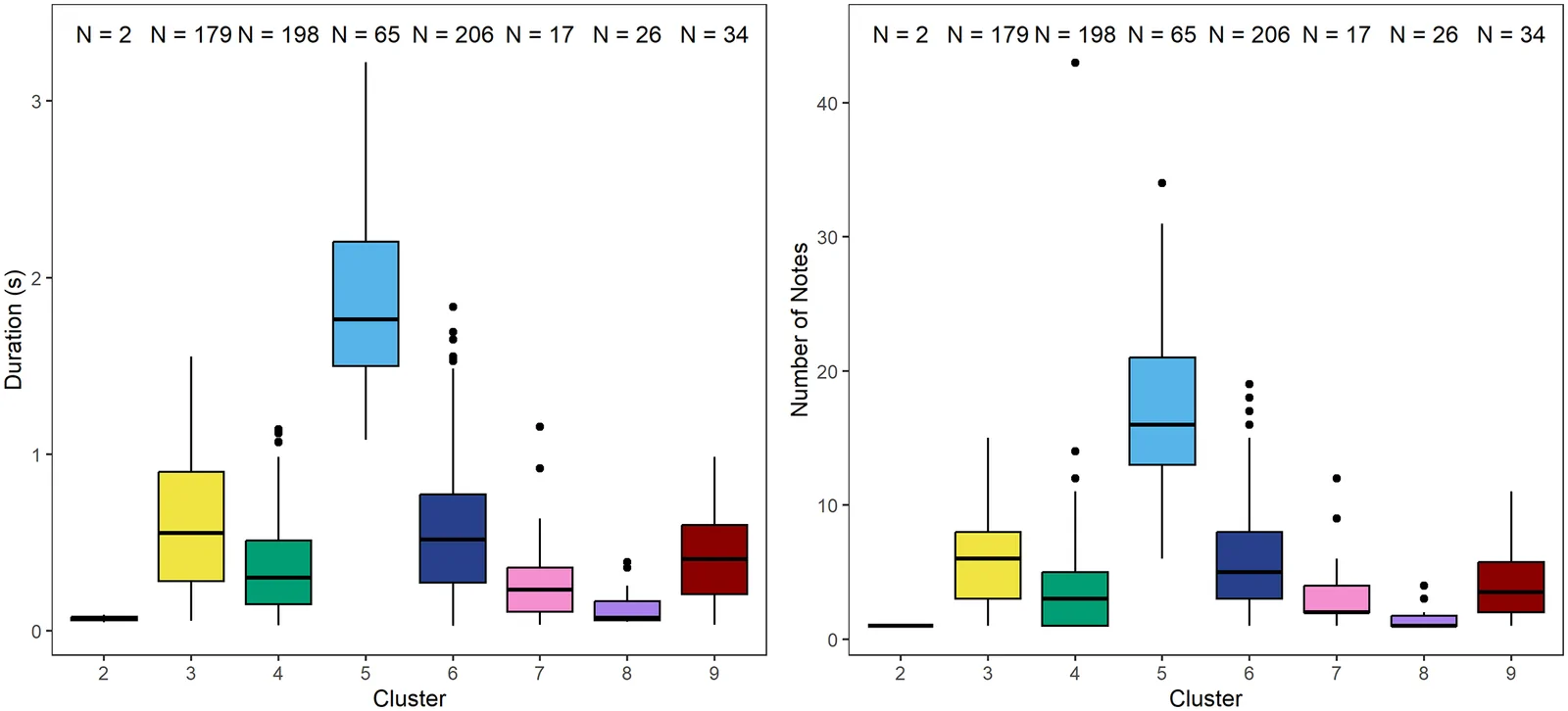
Metallic groans vary in the call duration (left) and the number of notes (right), with variation in these parameters being reflected in maximum fuzzy cluster memberships across clusters. Sample sizes are shown above each cluster.

#### Groans, grunts, and screeches.

Along with the metallic groan, Rüppell’s vultures issue several other calls in response to direct attack from conspecifics (Fig 10). These calls, like the metallic groans, had a very low overall typicality, with median TCs falling below the atypicality threshold. Therefore, calls given by individuals receiving aggression (metallic groans, groans, grunts, and screeches) are overall the least distinct and most graded calls in this vocal repertoire. First, screeches are sharp, noisy calls used mostly by juveniles and subadults in intense moments of trophic competition (S16 Audio in S1 File). Screeches can also be combined in sequence with the metallic groan syllables (S17 Audio in S1 File), generating a hybrid call relating to intense aggression over food. Groans are another call uttered only during trophic competition by all age classes (S18 Audio in S1 File). In contrast to the previously described calls given in response to aggression, grunts present as single note calls with a low frequency and are also emitted during less intense conflicts (S19 Audio in S1 File), most often during minor space aggression (54.5%), food competition (39.4%), and during stressful contexts including veterinary procedures (6.1%).

**Fig 10:**
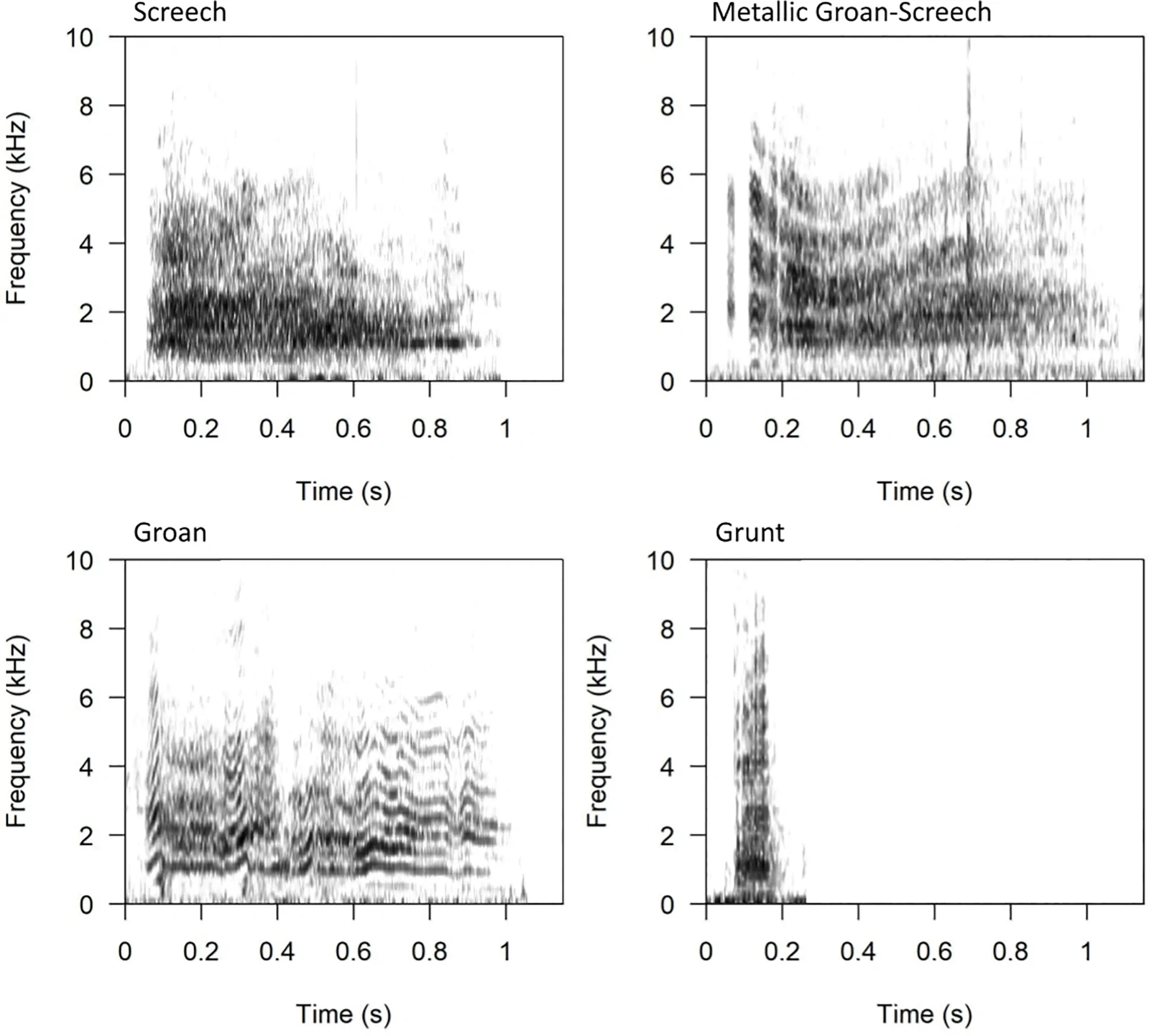
The diverse structures of several submissive and protest calls used by Rüppell’s vultures when receiving aggression from conspecifics. Juvenile and subadult individuals use screeches and metallic groan (MG)-screeches, while groans and grunts are produced across all age classes, excluding chicks.

#### Sex groans.

During copulations, the male mounted on their partner produced highly distinct sex groans repeatedly during a copulation bout (Fig 11; S20 Audio in S1 File), which had a relatively typical structure with 83.9% assigned to Cluster 7. These calls are relatively hoarse, low in frequency and loud, audible from at least 20 metres away. Across all 274 sex groans, 54.01% were classified as typical, 15.33% as intermediate, and 30.66% as atypical with a median TC of 0.56 (IQR = 0.61). These vocalisations are repeatedly produced throughout a copulation bout, exclusively by the individual mounting a conspecific (the male). We have no recordings of copulation calls produced by females during the rare instances in which they mount males.

**Fig 11:**
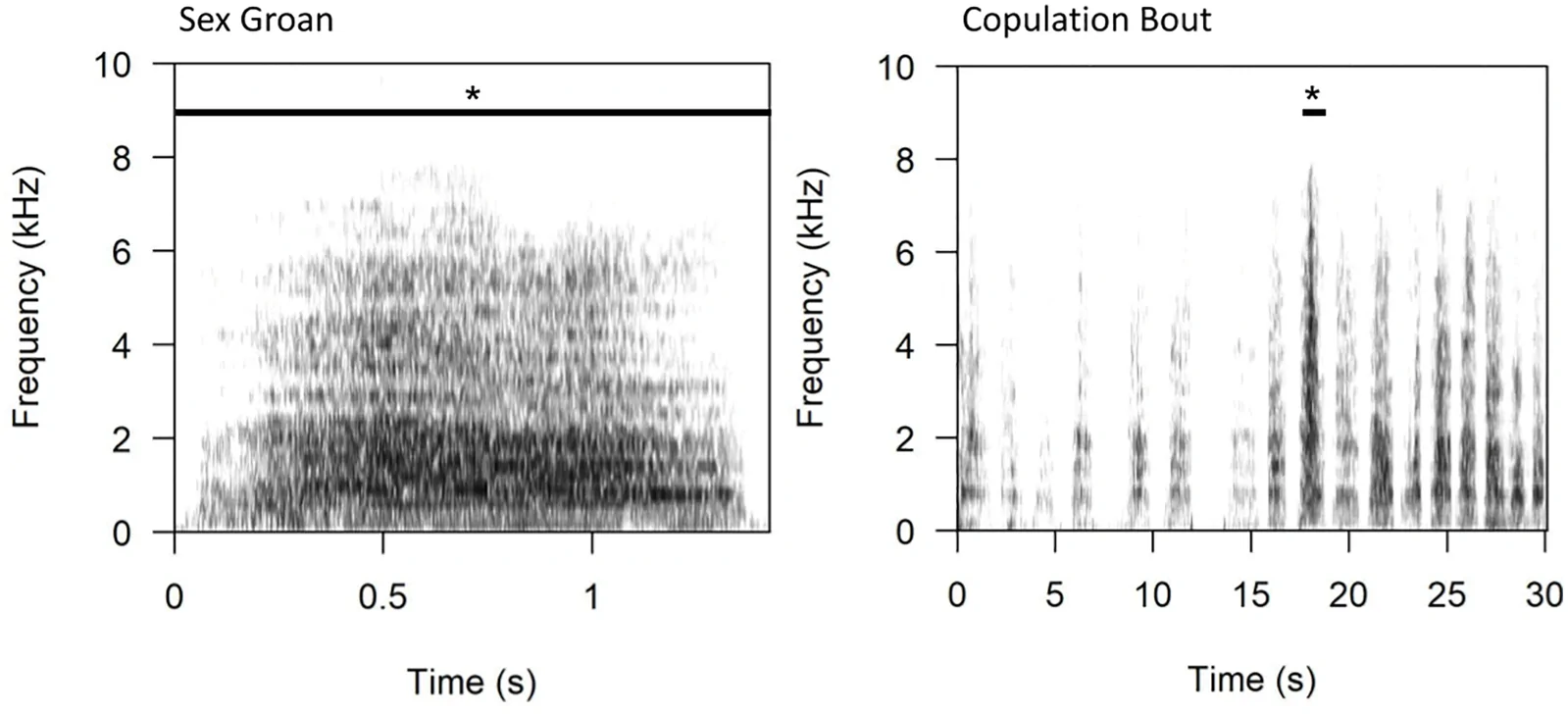
Sex groans (left) are produced during copulation, where many calls are repeated throughout a copulation bout (right). The sex groan shown on the left corresponds to the call indicated by * in the right panel. Copulation bouts can be as short as one or two sex groans, or last as long as several minutes. Note that the time scales differ between panels.

#### Small groan.

The small groan is a constant, low-amplitude vocalisation detected only three times during this study (S21 Audio in S1 File); however, because it is a low-amplitude call, it is likely underreported (Fig 12). Nevertheless, they clustered consistently into Cluster 7. All observed instances occurred with the caller’s beak closed, making caller identification difficult. These vocalisations were exclusively produced in non-agonistic social contexts, typically when multiple individuals were perched or resting on the ground.

**Fig 12:**
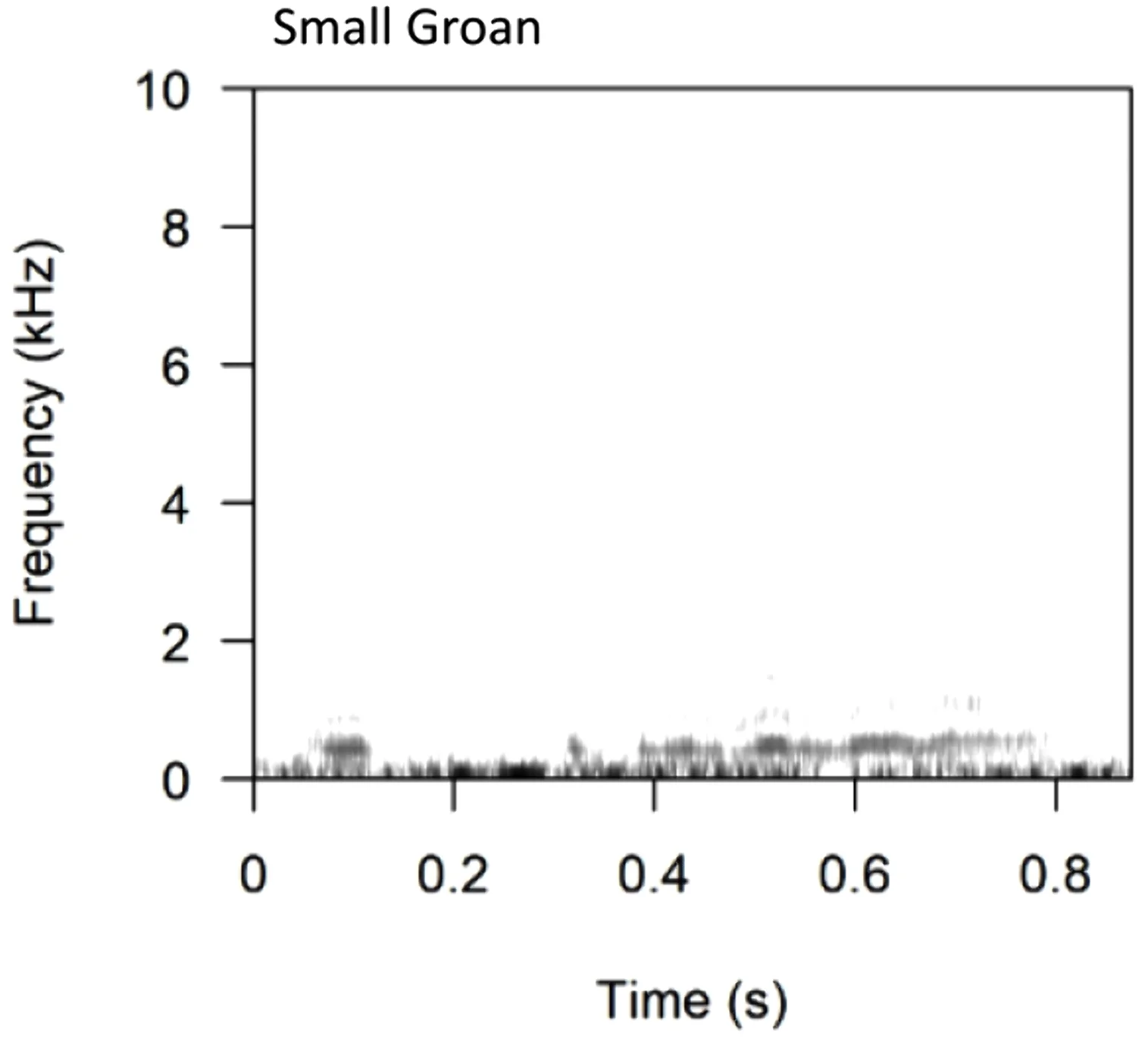
Faint spectrogram of the rare small groan call type characterised by a low energy continuous tonal sound below 1 kHz.

## Discussion

Social complexity has been proposed as a key evolutionary driver of communicative complexity [27,28], yet empirical support remains mixed within a limited set of model species. Expanding the taxonomic breadth of species used to test hypotheses on the evolution of communication is therefore essential, although this first requires substantial foundational work in non-model systems. To facilitate this, we characterised the vocal repertoire of the socially complex Rüppell’s vulture [90], building on previous work in griffon vultures [89]. This extends the growing evidence that Accipitrid vultures represent a promising system for investigating the evolution of avian communication, complementing established model taxa such as corvids, psittacines, and passerines [43,117–120].

Like griffon vultures, Rüppell’s vultures use a diverse array of vocalisations across a range of social contexts, with strong similarities in the repertoires of the two closely related species. This pattern is most parsimoniously explained by the hypothesis that the complex vocal system in these species evolved in a highly social common ancestor and has been retained in both lineages, reflecting the expectations of the SCHCC. Notably, our findings also highlight substantial gradation in several call types. While this gradation renders defining a discrete number of call types challenging, our results reveal that the calls of Rüppell’s vultures serve at least eight broad social contexts: receive aggression (metallic groans, screeches, grunts, groans), demand access (brays and bray-hisses), guarding threats (hisses and whistle-hisses), sexual behaviour (sex groans), non-agonistic social proximity (small groans), parent-chick relation (begging screeches and provision calls), chick general (chick calls), and pre-hatching within-egg vocalisations. The range of call types documented in this study indicates that Rüppell’s vultures possess a highly complex vocal repertoire, on par or exceeding that of the griffon vulture [89]. This therefore places the Rüppell’s vulture vocal repertoire well above the average call diversity among birds [47], being comparable to several highly social primates [49,121–124], and significantly more complex than those of many other colony-nesting non-passerines [98,100,125], and raptors [126].

In many taxa with complex vocal repertoires, vocal ontogeny typically begins with an early phase of exploratory vocalisations, with adult call types emerging incrementally before culminating in the mature repertoire [127]. For example, extensive research has revealed that early vocalisations and babbling in infant humans are critical for later language development [128,129], while similar vocal development trajectories have been documented in other non-human primates, cetaceans, and songbirds [127,130]. As our results reveal a complex vocal repertoire in Rüppell’s vultures, examining how these call types emerge over an individual’s lifetime is warranted [130]. Even though the chick calls are likely to be produced in a much smaller vocal tract with different resonance properties and membrane thickness, we did identify structural similarities between the calls of chicks (both pre- and post-hatching) and the syllables comprising metallic groans (see Figs 4 and 8). Given the highly combinatorial and complex nature of metallic groans, the stochastic vocalisations produced by chicks may resemble babbling, an important early stage of vocal development in complex communication systems, during which young individuals practice and explore the structure of their vocal repertoire before mastering its use as a functional signalling system. Additionally, the begging calls of young vultures had the highest typicality in the vocal repertoire, which may reflect their relatively simple social function of soliciting food from parents. Begging calls are also acoustically similar to the screeches and brays produced during trophic competition (both being harsh, broadband calls; see Figs 4, 7, and 10), suggesting a potential developmental link between these vocal signals and food-related contexts. Similar begging calls have also been documented in Cape vultures, produced by young birds within the cliff colony when their parents return, suggesting they also recognise their own parents and communicate accordingly [131].

After leaving the nest, individual Rüppell’s vultures become integrated into a stable social network with relatively clear dominance relations [90], where individuals need to manage several social ties and diverging interests. These factors increase competition among conspecifics when individuals are contesting limited resources. Direct physical aggression can resolve disputes but carries high energetic costs and risks of injury. Therefore, in complex social groups where individuals frequently compete for communal resources, cognitive strategies that mitigate these costs are advantageous. The evolution of advanced cognition is often linked to navigating social conflicts and manipulating competitors, a concept known as Machiavellian intelligence [2]. Consistent with this idea, Rüppell’s vultures appear to use several calls, primarily threats, protests and submissive signals, that allows them to optimise fitness during disputes, whether acting as aggressors or victims.

Threats serve to manipulate the behaviour of conspecifics to reduce the cost of conflict while increasing the benefits gained by the sender. Therefore, for threat signals to evolve, several authors argue they must reliably convey honest information about the caller’s aggressive intent or fighting ability [132–134]. Here, we identified two broad classes of threat signals produced by Rüppell’s vultures in two distinct contexts: resource defence and resource challenge. When defending a resource, individuals typically emit soft, atonal hisses and whistle-hisses, whereas brays and bray-hisses are more commonly produced during direct challenges against conspecifics already in possession of a resource. These signals likely convey the caller’s competitive intent or level of aggression and may function as Machiavellian signals that manipulate conspecifics’ behaviour to the caller’s advantage [2]. Both sets of signals exhibited an intermediately graded structure, with within-call variation potentially encoding information about the signaller’s motivation, dominance status, identity, or other factors. Notably, brays and bray-hisses had lower overall typicality than hisses and whistle-hisses. Future research should investigate whether these threats might also serve deceptive functions, with individuals potentially bluffing to gain access to resources they would otherwise lose in a direct physical contest [135]. Although beyond the scope of this study, hisses, whistle-hisses, brays, and bray-hisses were frequently accompanied by visual displays, including changes in neck, wing, and body posture, as well as slow foot-stomping movements (see S1 Video in S1 File). Taken together, these audio-visual displays have previously been described as highly ritualised [89,136], suggesting they may rely on relatively simple cognitive processes. To better understand the socio-cognitive abilities of this species, future research should investigate how social context shapes the production of these multimodal signals and whether individuals adjust them in response to the behaviour of receivers.

If threats fail to avert contests or conflicts escalate into physical attacks, the risk of injury rises sharply, making effective conflict resolution crucial. Vulture skeletons are optimised for soaring flight, being lightweight and hollow, and are therefore particularly vulnerable during dense feeding aggregations [136]. This underscores the potential importance of vocal signalling in mitigating costly conflicts. As predicted, we found diverse, finely graded call types, which the vultures use when receiving aggression from conspecifics, comprising the most complex functional call class. This class includes metallic groans, screeches, grunts, and groans, all characterised by low overall typicality, high within-call variation, and extreme acoustic gradation between call types. This contrasts with the more distinct structure of the threat vocalisations. Among these graded submissive calls, the metallic groan was by far the most frequently observed. Our findings, along with those in the griffon vulture [89], converge on the conclusion that the metallic groans contain complex acoustic properties previously documented in the vocal systems of intelligent, large-brained mammals, including primates, elephants and cetaceans [30–32,89]. Metallic groans are combinatorially structured, incorporating variable, rapidly pronounced syllables with non-linear phenomena while modulating pulse rates, potentially allowing the caller to convey fine-grained biologically meaningful information (e.g., identity, emotion, motivation). Contrary to Romani et al. [89], who reported its occurrence across broader social contexts, we found metallic groans to be specifically associated with receiving aggression. We therefore interpret the metallic groan as a possible submissive or protest vocalisation, with its gradation potentially allowing individuals to encode information about arousal, motivational state, and intent to de-escalate conflict. Although much is still unknown about how these vultures apply metallic groans across diverse social contexts [89], these calls constitute a complex and flexible communication system in the *Gyps* vultures, and further studies should explore its complexity.

Our results reveal substantial variation in the call duration and the number of notes depending on maximum cluster assignment. These findings raise the question of whether gradation in metallic groans reflects variation in conflict intensity and serves an adaptive communicative function. In more prolonged or intense conflicts, one might expect recipients of aggression to produce longer, louder, and higher-frequency calls to more effectively signal distress or submission [63]. Variation within the metallic groans may also be biologically meaningful, sharing information about motivation or arousal and differ depending on the contest intensity, underscoring the need for more detailed analyses of the constituent parts of metallic groans. Romani et al. [89] reported that elements such as non-linear noise gaps and regular pulsing within syllables differ depending on the agonistic nature of the context. They further report that fewer syllables are used during direct attack and aggression, which may make signals clearer and faster in high-risk interactions [64]. Overall, we find that metallic groans vary along at least two primary dimensions: the number of notes used and the structure of the syllables they are composed of. Consequently, the metallic groan remains a promising candidate for investigating how acoustic gradation and sound sequences encode social and emotional information in *Gyps* vultures. It also remains unclear in which social contexts the other protest and submissive calls, namely screech, metallic groan-screech, groan, and grunt are used. Therefore, future research should examine how call structure relates to conflict intensity and rank difference between aggressor and victim.

Alongside social factors, predation risk is a major ecological pressure linked to vocal complexity [137,138]. While many species use complex, referential alarm-calling systems (e.g.[59,139,140],), our findings, together with those on griffon vultures [89], suggest that *Gyps* vultures do not produce alarm calls, likely because adult vultures face no natural predators. Furthermore, although some potential nest predators exist (e.g., large raptors and corvids, snakes, and baboons), the species’ steep cliff nesting sites are largely inaccessible to most terrestrial predators, substantially reducing predation pressure on eggs and chicks [141]. Nonetheless, given their tendency to forage near potentially dangerous mammalian carnivores, it is plausible that they have evolved alternative risk-mitigation strategies, such as gaze cueing or visual signals, or that in these contexts they may employ certain calls also used in intraspecific interactions (e.g., hisses). Exploring these possibilities may provide an interesting direction for future research.

Understanding the vocalisations associated with reproduction is particularly important for this critically endangered species [142], as such knowledge may directly inform captive breeding and reintroduction efforts. Birds of prey copulate more frequently and conspicuously than most other avian taxa, with males often vocalising during these events [143]. In Cape vultures for example, pairs copulate on average 100 times per clutch, and the copulation period extends from 90 days before to 140 days after clutch laying [82], and a similarly protracted mating pattern is seen in black-winged kites (*Elanus caeruleuas*) [144]. While the primary function of copulation is certainly fertilisation, and a single copulation is often sufficient to achieve this, the unusually frequent mating observed in raptors raises questions about potential secondary functions. Multiple hypotheses have been proposed to explain the conspicuous and frequent copulatory behaviour in vultures, including male-male competition (with potential extra-pair copulations), female mate choice, pair-bonding, or territory signalling, with the latter receiving modest support [145]. The territory signalling hypothesis states that, because access to high-quality nesting sites is often a limiting factor in raptor population expansion, copulations have evolved a secondary function to advertise a pair’s sexual activity to defend a nesting territory [143]. Our results suggest that sex groans in Rüppell’s vultures are acoustically distinct calls, yet with some gradation and variation within this call type (46% classed as intermediate or atypical) and tend to be audible from at least 20 meters away, meaning they are detectable from neighbouring nests (based on the hearing abilities reported in [80]). Given that there is some variation in this call type, investigating whether these differences encode important information about the sender (e.g., identity, size, age) will provide further insights into this call’s function. Discriminating between these possibilities will require targeted playback experiments and long-term behavioural monitoring of copulation frequency across the breeding season [145].

In addition to the sex groans, another call type holds significant potential for these programmes: the pre-hatching call (see Video S2 in S1 File). Captive breeding of Rüppell’s vultures has proven challenging, especially with artificial incubation protocols. In many zoos, the egg is removed for machine incubation and replaced with a replica, and parents only receive their chick several days after hatching: a husbandry procedure which increases reproductive success [107]. This delay may however be problematic, particularly for inexperienced parents, who may fail to recognise or accept their offspring, potentially due to the absence of pre-hatching vocalisations, which in many species serve as important cues for triggering parental behaviour. Evidence from studies on precocial birds such as gulls and terns, as well as crocodilians, shows that pre-hatching vocalisations can significantly influence parental responsiveness or influence hatching behaviour [146–148]. Therefore, a priority for future research should be to determine the function of these pre-hatching calls and hereafter developing technology to replicate this to improve chick acceptance and survival. This would offer a practical approach to support the success of captive breeding programmes for this critically endangered species [149,150].

This study represents another step in establishing vultures as a promising model system for researching the social complexity hypothesis for communicative complexity. Our findings indicate that the vocal communication of Rüppell’s vultures closely resembles that of the closely related griffon vulture [89], with both species possessing similarly large vocal repertoires. Notably, the repertoire size in both species falls on the upper end of vocal complexity across a wide range of avian lineages [47]. We note, however, that our study was conducted in captivity, which likely limits the expression of certain behavioural contexts and, consequently, the production of associated vocalisations. It is therefore possible that additional call types occur in wild populations, meaning the repertoire described here may represent a conservative estimate of the species’ full vocal repertoire. For *Gyps* vultures, one important context absent from captivity is the movement interactions that occur during soaring flight. In the wild, they routinely travel in groups and rely on aerial information networks when making foraging decisions while soaring at high altitudes [81], creating opportunities for social interactions that do not occur in captive settings. If vocal communication contributes to coordinating these movements or maintaining group cohesion during flight, additional context-specific vocalisations may exist that were not captured in the present study.

While this study is not comparative and therefore cannot directly support a theory on the evolutionary processes affecting communication, our data are consistent with the SCHCC. Armed with evidence that the Rüppell’s vulture and griffon vulture use complex sound signalling systems to navigate the challenges imposed by colony living and highly social foraging strategies [76,82,90], considerable research effort into vulture communication could generate important insights into the evolution of cognition and communication. Therefore, researchers should continue to characterise the vocal repertoire of other vulture species differing in their social complexity to test whether social and vocal complexity share evolutionary trajectories across these species.

Furthermore, while vultures can offer critical insights into cognitive evolution, their rapidly declining populations present an urgent conservation challenge, with Rüppell’s vulture having lost an estimated 97% of their population over the past three generations [151]. As the most threatened group of birds globally, widespread vulture extinction could trigger severe ecological consequences including trophic cascades, disease spread, pest outbreaks, and increased greenhouse gas emissions [152,153]. Significantly, the value of ecosystem services provided by vultures in Southern Africa alone is an estimated $1.8 billion per year, stressing the urgent need for their preservation as a critical international concern [154]. Therefore, studying the complex social lives and communication of vultures will not only advance our understanding of cognition but also directly contribute towards improving their conservation efforts, which could yield substantial economic returns [76,149,150,154].

## Supporting information

S1 File Supplemental audio and video files. (ZIP)

S1 Fig Silhouette values per clustering solution. Fuzzy silhouette values for each combination of k and μ tested up to μ = 1.5 (μ values greater than 1.5 yielded unstable cluster solutions with unclear membership values). The nine-cluster solution peaked at μ = 1.4 and was also among the best number of clusters across other values of μ.
(TIFF)

S2 Fig Eight cluster results. Visualisation of Rüppell’s vulture vocal repertoire structure based on an eight cluster solution with a μ value of 1.4. Data are presented in the same manner as Fig 2.
(TIFF)

S3 Fig Ten cluster results. Visualisation of Rüppell’s vulture vocal repertoire structure based on a ten cluster solution with a μ value of 1.4. Data are presented in the same manner as Fig 2.
(TIFF)

S1 Table Study population. Overview of the individual vultures in our study population, including demographic information.
(XLSX)

S2 Table Call parameters. List of the 38 acoustic parameters and their definitions as used in this study.
(XLSX)

S3 Table Summary of call parameters per call type. Mean and standard deviation of all 38 acoustic parameters per call type.
(XLSX)

S4 Table Fuzzy cluster memberships. Overview of the distribution of each call type across fuzzy clusters.
(XLSX)

S5 Table Typicality summary. Overview of the mean, standard deviation, median, and inter-quartile range of the typicality coefficients per call type. Additionally, this table includes the proportion of each call type that are classed as typical, intermediate, and atypical.
(XLSX)

S6 Table Behavioural contexts. Overview of the distribution of calls across behavioural contexts.
(XLSX)

S7 Table Metallic groan variation. Summary of between over within cluster variation in each acoustic parameter in metallic groans.
(XLSX)
