# Supplementary material for: The complex, graded vocal repertoire of the social Rüppell’s vulture
Source: PLoS One. 2026 Oct 21;21(10):e0360246. doi: 10.1371/journal.pone.0360246 (PMC13686281; doi:10.1371/journal.pone.0360246)
Supplement: Supplementary file 1 [file pone.0360246.s012.docx]

This paper addresses the interesting topic of describing the vocal repertoire of Rüppell’s vulture, a highly social raptor species belonging to the *Gyps* genus. Non passerine bird species have been neglected in the study of vocal behaviour in comparison to passerines, leading to the bulk of knowledge about this behaviour being skewed and non representative of the whole clade. Therefore studies like the one at hand are timely and necessary.

The manuscript at hand is overall very well written, coherent, with a good flow that is easy to follow. The theoretical introduction, methods and analyses, results and their discussion are all scientifically sound and address the investigations outlined by the authors. I discuss the following points to highlight some ambiguities and questions as a reader, hoping that the authors will find them useful.

**Genearl comments:**

- My main questions deal with the theoretical motivation for this study. The authors cite the literature correlating relative brain size to increased cognitive functions, and propose a link to vocal complexity. Increased encephalisation has only been linked to higher brain functions in primates as the authors pointed out, but was found to be a poor measure of the same cognitive abilities in Aves, because the neural architecture is different when comparing the vocal learning centres of the two clades, see Bolhuis et al 2010 doi:10.1038/nrn2931
- The second line of theoretical motivation behind the study is the Social Complexity Hypothesis for Communicative Complexity. There are some points I would like to raise here as well. For example, in line 17: I am rather confused by the reasoning behind the statement “Although supported in specific phylogenetic lineages, evidence for the SCHCC is often inconsistent” then go on to argue that it “is drawn largely from a small number of well-studied taxa, and frequently fails to consider a species’ full vocal repertoire”. If the hypothesis is inconsistent, do the authors think it is because it relies on limited taxa and few of their calls only? The criticism of the hypothesis is largely based on the general use of the term “social complexity” without standardised metric. Leighton and Birmingham (2021), and Leighton (2017) attempted to address this critique by correlating vocal complexity, measured as functional repertoire size, to specific aspects of social complexity, like group size, group stability, length of social bond etc. They found that cooperative breeding in particular explained this measure of vocal complexity, but was not the only factor behind it, but rather that ecological factors like foraging strata, contributed to explaining it.
  I think therefore that the authors might want to consider that, since both the study species and the closely related Griffon vulture used for comparison are colonial breeders, this aspect of sociality could be the driver of social complexity in the species.
- How did the authors ascertain the captive conditions allowed for the production of the full repertoire? When captivity limits the full gamut of social contexts, particularly those that involve movement? Maybe emphasise this restriction of full natural behaviour imposed by captivity in the introduction and methods, because the introduction (Lines 211 – 213) currently argues for the importance of using the full vocal repertoire to study the evolution of a species communication system. Same applies for lines 326-329, I do not think it is accurate to state these contexts will result in the full gamut of the repertoire, seeing that they are captive and their main behaviour of movement via flying is not part of the study.
- Line 27- 28 “The Rüppell’s vulture repertoire exceeds the average repertoire size

observed across birds and is likely an adaptation to this species’ social life”, and Line 197 – 198 “12 distinct calls make it one of the largest repertoire sizes in birds” Maybe it is worth emphasising that you are talking here about non passerines, because song repertoires are on average much larger, particularly those of mimics. The Leighton and Birmingham, 2021 paper cited by the authors uses functional repertoire sizes, not absolute repertoire sizes, and reports only calls, not song types. the 12 – 16 call types found in this study are below average according to figure 1 of the cited paper.

- Method selection, how does the fuzzy k-means algorithm account for individual variation within the calls and the non independence of calls drawn from the same individual? when more than one call group together into a distinct cluster, how much variation is a mark of two different but graded call types of the same category, and what is the variation cutoff that signals the same call with individual variation among callers? For example, the chick and juvenile begging calls, the difference can either be the effect of age and development, but could also be the result of individual differences that persisted past developmental stages. In the Chen and Weins (2020) reference, it is stated that the within call type variation can be accounted for by the caller’s affective state, but there is a large body of literature that account for the within call variation by individual differences, either those that reflect morphological differences or markers of group membership among others. It is necessary to clarify this point in the introduction.
- Similar to the previous point, when considering hisses and whistle hisses, bray and bray hisses, MG and MG screechs, why assume they are different calls when it has a hiss or a screech component, and not just a syntactic combination of the call and a hiss or a call and screech?
- Finally, regarding the data availability, the authors stated that there are no restrictions on the data and that it is all readily available, but I found no link to the dataset or the R pipeline code. It is a good idea to include those along with the provided call exemplars for full transparency.

**Line specific comments**

- 279 – 285 – what about the prehatching parent calls? Do they vocalise to the unhatched eggs?
- Line 287 – 297 at what distance were these vocalisations recorded? And how did the experimenter decide at the appropriate distance given the various calls with different audible ranges?
- 288-293 are hyenas and snakes competitors only or predators? What are the predators of the vultures, particularly at the nest?
- Line 336 – 338 – what was the cutoff for the SNR?
- Figure 2 C colours are very faint for some calls, consider modifying the plot to maybe add border to the dots or replace the very light colours with darker shades.
- Line 425: “ In our analysis, we generated a single UMAP using the ‘umap’

function from the ‘uwot’ R package, and coloured the datapoints based on the subjective call labels determined by an observer”. Can you clarify the “subjective” nature of this labelling? It is relevant to how the reader will interpret figure 2.

- Following from the previous point: The call types in Figure 2, panel A and their names were not introduced prior to this point nor matched to the contexts in table 1. The first mention of call types was in line 351 stating the authors followed Romani et al 2002, but it did not mention the number of call types nor their names and contexts. it should be made clearer how good of a fit the observed calls in this study matched the cited study, and best to introduce the calls in table 1, matching them to context.
- Did the colouring of the datapoints in figure 2 panel A use the calls listed in Romani et al as a reference? If so, why did you call it “subjective” labelling, a descriptor that suggests the observer classified the different call types visually or audibly. Figure 3 introduces the call types and the matching contexts; it would be clearer if this is introduced earlier for the manual matching.
- Line 464-465 table S7 could not be found in the manuscript, I am guessing it provides the matched call types and contexts resulting from the Pearson’s *X^2^* test, but the “subjective” matching should have been introduced first, to drive home the point of this analysis
- Figure 3 and the *X*^2^ test results: the test assumes the calls to be independent samples, but I am assuming many of the calls came from the same individual or calling bout? If that is true then the effective sample size is smaller than the stated number of calls (pseudo replication), which will result in an inflated value of the test and its significance. The test also assumes sufficient expected counts for each cell, which is not the case as seen in the sparsity of some call types in figure 3. Perhaps using a linear modelling approach that controls for repeated measures would be more appropriate, and potentially collapsing some of the rare call categories into those that are acoustically and contextually similar.
- Line 566 typicality threshold. If the typicality coefficient Line 413 – 417 is below the lower typicality threshold, why did the authors consider it graded call? Is it because of the consistent contexts?
- Line 580 table S8 cannot be found in the manuscript
- Line 644 – 650, the opening sentence is not very informative here. Referring back to my first two points, maybe stating that hypothesis is contested without clarification of how your study addresses that is not a clear motivation for conducting the study.
- Lines 652 – 653 : a common ancestor is not the only mechanism by which related species can share an adaptation, and since the study does not look into how the vocal repertoire evolved, it is not necessary to make a statement about it.
- Lines 661 -663 : as mentioned above, the figure in Leighton and Birmingham does not support this statement.
- 681 – 683 – what are the spectral characteristics in question? And how do the authors link those to the function of the three call types?
- Lines 763 – 771 : did the authors explore the existence of nest alarm calling? Despite not having natural predators as adults, the young are still vulnerable at the nest, and it is an interesting point to mention if it has been researched.
