# Supplementary material for: The complex, graded vocal repertoire of the social Rüppell’s vulture
Source: PLoS One. 2026 Oct 21;21(10):e0360246. doi: 10.1371/journal.pone.0360246 (PMC13686281; doi:10.1371/journal.pone.0360246)
Supplement: Supplementary file 2 [file pone.0360246.s014.docx]

Dear Editor,

I have received the revised manuscript and reviewed the changes made, and I find the changes the authors made to effectively address the comments from the first round, resulting in a more replicable and accessible study. I do have minor follow up comments that I list below and hope the authors will find it useful.

General comment:

The introduction is very well written, concise and paints an interesting picture. I would however reorganise its parts to open with the link between social and communication complexity. The current first paragraph reads like it naturally follows from the second.

Specific comment:

- The *X*^2^ test results: The authors’ response regarding the low expected cell counts and how they relied on Monte Carlo simulated p-values sufficiently addresses my comment. My other concern regarding the non-independence of observations and how that violates the assumptions of the test, still stand. I appreciate that under the setup of data collection it was not possible to account for caller identity and that accordingly the analysis only serves an exploratory purpose. However, I do not agree that the current presentation of the results reflect this deviation from the test assumptions. The first half recommends the reader interpret the p-values cautiously, while the second half affirms the results to reveal “clear, non-random structuring of call use across behavioural contexts. This phrasing, along with the first two lines presenting the result assert a stronger claim than supported by the results of the *X*^2^ test, making the two statements presenting this result contradictory. The authors can still state the data descriptively show a strong distinction of call type production within context, without making an unsupported statement about statistical inference. The observed pattern of call production within context can be skewed to a higher contribution of a large number of calls belonging to one individual for example, without such pattern being detected by the test.
