# Supplementary material for: The complex, graded vocal repertoire of the social Rüppell’s vulture
Source: PLoS One. 2026 Oct 21;21(10):e0360246. doi: 10.1371/journal.pone.0360246 (PMC13686281; doi:10.1371/journal.pone.0360246)
Supplement: Supplementary file 4 [file pone.0360246.s016.docx]

Dear Dr. Brenton Cooper,

We sincerely appreciate the time and effort you and the reviewer have dedicated to evaluating our manuscript titled "The complex, graded vocal repertoire of the social Rüppell’s vulture”. We appreciate the opportunity to revise our manuscript and address the reviewer’s two comments.

Below, we provide detailed responses to each point raised during the review process. For clarity and ease of reference, we have reproduced the comments from the decision letter and included our responses directly beneath each one in blue. To facilitate navigation, all editor and reviewer comments have been numbered. As requested, all revisions have been made using track changes, and the corresponding line numbers in the track changes revised manuscript are indicated in red below; all changes are also visible in the tracked-changes copy.

**Comments and Responses**

**Reviewer 1**

**Comment 1**: The introduction is very well written, concise and paints an interesting picture. I would however reorganise its parts to open with the link between social and communication complexity. The current first paragraph reads like it naturally follows from the second.

***Response****: We thank the reviewer for this comment and appreciate their positive assessment of the introduction. We have carefully considered the suggested reorganisation; however, we have retained the current structure because we feel it provides a more logical progression from the broader theoretical context of the coevolution of sociality and intelligence to the more specific role of communication as an important social-cognitive domain through which this relationship can be tested. We believe this structure best reflects the theoretical rationale and aims of our study.*

**Comment 2**: The X2 test results: The authors’ response regarding the low expected cell counts and how they relied on Monte Carlo simulated p-values sufficiently addresses my comment. My other concern regarding the non-independence of observations and how that violates the assumptions of the test, still stand. I appreciate that under the setup of data collection it was not possible to account for caller identity and that accordingly the analysis only serves an exploratory purpose. However, I do not agree that the current presentation of the results reflect this deviation from the test assumptions. The first half recommends the reader interpret the p-values cautiously, while the second half affirms the results to reveal “clear, non-random structuring of call use across behavioural contexts. This phrasing, along with the first two lines presenting the result assert a stronger claim than supported by the results of the X2 test, making the two statements presenting this result contradictory. The authors can still state the data descriptively show a strong distinction of call type production within context, without making an unsupported statement about statistical inference. The observed pattern of call production within context can be skewed to a higher contribution of a large number of calls belonging to one individual for example, without such pattern being detected by the test.

***Response****:*

*We thank the reviewer for this comment and agree that, despite our intention to use the χ² analysis descriptively, the potential non-independence of observations limits the validity of its statistical inference. We have therefore removed the χ² test and associated p-value from the manuscript and now present the relationship between call types and behavioural contexts descriptively (based on the distribution show in Figure 3 and Table S6).*

*We have removed text relating to this statistical test in the Methods L403 and the Results L442.*
