# Supplementary material for: The complex, graded vocal repertoire of the social Rüppell’s vulture
Source: PLoS One. 2026 Oct 21;21(10):e0360246. doi: 10.1371/journal.pone.0360246 (PMC13686281; doi:10.1371/journal.pone.0360246)
Supplement: Peer review history

# Peer review: Decision Letter 0

**DOI:** 10.1371/journal.pone.0360246.r001

**Authors:** Brenton Cooper

4 Jun 2026

Dear Dr. Proudfoot,

Please submit your revised manuscript by Jul 19 2026 11:59PM. If you will need more time than this to complete your revisions, please reply to this message or contact the journal office at plosone@plos.org. When you're ready to submit your revision, log on to https://www.editorialmanager.com/pone/ and select the 'Submissions Needing Revision' folder to locate your manuscript file.

- A letter that responds to each point raised by the academic editor and reviewer(s). You should upload this letter as a separate file labeled 'Response to Reviewers'.
- A marked-up copy of your manuscript that highlights changes made to the original version. You should upload this as a separate file labeled 'Revised Manuscript with Track Changes'.
- An unmarked version of your revised paper without tracked changes. You should upload this as a separate file labeled 'Manuscript'.

If you would like to make changes to your financial disclosure, please include your updated statement in your cover letter. Guidelines for resubmitting your figure files are available below the reviewer comments at the end of this letter.

If applicable, we recommend that you deposit your laboratory protocols in protocols.io to enhance the reproducibility of your results. Protocols.io assigns your protocol its own identifier (DOI) so that it can be cited independently in the future. For instructions see: https://journals.plos.org/plosone/s/submission-guidelines#loc-laboratory-protocols. Additionally, PLOS ONE offers an option for publishing peer-reviewed Lab Protocol articles, which describe protocols hosted on protocols.io. Read more information on sharing protocols at https://plos.org/protocols?utm_medium=editorial-email&utm_source=authorletters&utm_campaign=protocols.

As the corresponding author, your ORCID iD is verified in the submission system and will appear in the published article. PLOS supports the use of ORCID, and we encourage all coauthors to register for an ORCID iD and use it as well. Please encourage your coauthors to verify their ORCID iD within the submission system before final acceptance, as unverified ORCID iDs will not appear in the published article. *Only*  the individual author can complete the verification step; PLOS staff *cannot*  verify ORCID iDs on behalf of authors.

We look forward to receiving your revised manuscript.

Kind regards,

Brenton G. Cooper, Ph.D.

Academic Editor

PLOS One

Journal Requirements:

When submitting your revision, we need you to address these additional requirements.

1. Please ensure that your manuscript meets PLOS ONE's style requirements, including those for file naming. The PLOS ONE style templates can be found at

https://journals.plos.org/plosone/s/file?id=wjVg/PLOSOne_formatting_sample_main_body.pdf and https://journals.plos.org/plosone/s/file?id=ba62/PLOSOne_formatting_sample_title_authors_affiliations.pdf

2. To comply with PLOS One submissions requirements, in your Methods section, please provide additional information regarding the experiments involving animals and ensure you have included details on (1) methods of sacrifice, (2) methods of anesthesia and/or analgesia, and (3) efforts to alleviate suffering.

3. When completing the data availability statement of the submission form, you indicated that you will make your data available on acceptance. We strongly recommend all authors decide on a data sharing plan before acceptance, as the process can be lengthy and hold up publication timelines. Please note that, though access restrictions are acceptable now, your entire data will need to be made freely accessible if your manuscript is accepted for publication. This policy applies to all data except where public deposition would breach compliance with the protocol approved by your research ethics board. If you are unable to adhere to our open data policy, please kindly revise your statement to explain your reasoning and we will seek the editor's input on an exemption. Please be assured that, once you have provided your new statement, the assessment of your exemption will not hold up the peer review process.

4. We note that the grant information you provided in the ‘Funding Information’ and ‘Financial Disclosure’ sections do not match.

When you resubmit, please ensure that you provide the correct grant numbers for the awards you received for your study in the ‘Funding Information’ section.

5. Thank you for stating the following in the Acknowledgments Section of your manuscript:

We are grateful to all who supported this research, including Zofia Trafas, Fleur Breeveld, Noor Ooms, and all members of the Vulture Cognition Lab and the Animal Behaviour and Cognition department at Utrecht University for the many insightful discussions that enriched this project. Special thanks go to Joost Lammers from Vogelpark Avifauna for granting access to their population of Rüppell’s vultures, and to the dedicated bird caretakers at both Diergaarde Blijdorp and Vogelpark Avifauna for their assistance, particularly Rolf Ordelman for facilitating our recordings of chicks and eggs during incubation. Finally, we extend our appreciation to Kurt Hammerschmidt for his guidance with fuzzy clustering, and to Tim-Joshua Andres and Arja Mentink for their valuable input on implementing our analyses. Financial support for this study was provided by the Ministry of Education, Culture and Science of the Netherlands (a starters and incentive grant #U2023-169 to J.J.M.M.), and from the Dobberke Foundation for Comparative Psychology, the Netherlands (KNAWWF|DA|3211|022 to A.M.S.).

We note that you have provided funding information that is not currently declared in your Funding Statement. However, funding information should not appear in the Acknowledgments section or other areas of your manuscript. We will only publish funding information present in the Funding Statement section of the online submission form.

Please remove any funding-related text from the manuscript and let us know how you would like to update your Funding Statement. Currently, your Funding Statement reads as follows:

Financial support for this study was provided by the Ministry of Education, Culture and Science of the Netherlands (a starters and incentive grant #U2023-169 to J.J.M.M.), and from the Dobberke Foundation for Comparative Psychology, the Netherlands (KNAWWF|DA|3211|022 to A.M.S.).

Please include your amended statements within your cover letter; we will change the online submission form on your behalf.

6. Please amend your list of authors on the manuscript to ensure that each author is linked to an affiliation. Authors’ affiliations should reflect the institution where the work was done (if authors moved subsequently, you can also list the new affiliation stating “current affiliation:….” as necessary).

7. We note that Figure 1 in your submission contain copyrighted images. All PLOS content is published under the Creative Commons Attribution License (CC BY 4.0), which means that the manuscript, images, and Supporting Information files will be freely available online, and any third party is permitted to access, download, copy, distribute, and use these materials in any way, even commercially, with proper attribution. For more information, see our copyright guidelines: http://journals.plos.org/plosone/s/licenses-and-copyright.

We require you to either (1) present written permission from the copyright holder to publish these figures specifically under the CC BY 4.0 license, or (2) remove the figures from your submission:

a. You may seek permission from the original copyright holder of Figure 1 to publish the content specifically under the CC BY 4.0 license.

We recommend that you contact the original copyright holder with the Content Permission Form (http://journals.plos.org/plosone/s/file?id=7c09/content-permission-form.pdf) and the following text:

“I request permission for the open-access journal PLOS ONE to publish XXX under the Creative Commons Attribution License (CCAL) CC BY 4.0 (http://creativecommons.org/licenses/by/4.0/). Please be aware that this license allows unrestricted use and distribution, even commercially, by third parties. Please reply and provide explicit written permission to publish XXX under a CC BY license and complete the attached form.”

Please upload the completed Content Permission Form or other proof of granted permissions as an "Other" file with your submission.

In the figure caption of the copyrighted figure, please include the following text: “Reprinted from [ref] under a CC BY license, with permission from [name of publisher], original copyright [original copyright year].”

b. If you are unable to obtain permission from the original copyright holder to publish these figures under the CC BY 4.0 license or if the copyright holder’s requirements are incompatible with the CC BY 4.0 license, please either i) remove the figure or ii) supply a replacement figure that complies with the CC BY 4.0 license. Please check copyright information on all replacement figures and update the figure caption with source information. If applicable, please specify in the figure caption text when a figure is similar but not identical to the original image and is therefore for illustrative purposes only.

8. We notice that your supplementary [figures] are included in the manuscript file. Please remove them and upload them with the file type 'Supporting Information'. Please ensure that each Supporting Information file has a legend listed in the manuscript after the references list.

9. Please include captions for your Supporting Information files at the end of your manuscript, and update any in-text citations to match accordingly. Please see our Supporting Information guidelines for more information: http://journals.plos.org/plosone/s/supporting-information.

10. If the reviewer comments include a recommendation to cite specific previously published works, please review and evaluate these publications to determine whether they are relevant and should be cited. There is no requirement to cite these works unless the editor has indicated otherwise.

[Note: HTML markup is below. Please do not edit.]

Reviewers' comments:

Reviewer's Responses to Questions

**Comments to the Author**

1. Is the manuscript technically sound, and do the data support the conclusions?

Reviewer #1: Yes

**********

2. Has the statistical analysis been performed appropriately and rigorously?

Reviewer #1: Yes

**********

3. Have the authors made all data underlying the findings in their manuscript fully available?

The PLOS Data policy

Reviewer #1: Yes

**********

4. Is the manuscript presented in an intelligible fashion and written in standard English?

Reviewer #1: Yes

**********

Reviewer #1: This paper addresses the interesting topic of describing the vocal repertoire of Rüppell’s vulture, a highly social raptor species belonging to the Gyps genus. Non passerine bird species have been neglected in the study of vocal behaviour in comparison to passerines, leading to the bulk of knowledge about this behaviour being skewed and non representative of the whole clade. Therefore studies like the one at hand are timely and necessary.

The manuscript at hand is overall very well written, coherent, with a good flow that is easy to follow. The theoretical introduction, methods and analyses, results and their discussion are all scientifically sound and address the investigations outlined by the authors. I discuss the following points to highlight some ambiguities and questions as a reader, hoping that the authors will find them useful.

Genearl comments:

• My main questions deal with the theoretical motivation for this study. The authors cite the literature correlating relative brain size to increased cognitive functions, and propose a link to vocal complexity. Increased encephalisation has only been linked to higher brain functions in primates as the authors pointed out, but was found to be a poor measure of the same cognitive abilities in Aves, because the neural architecture is different when comparing the vocal learning centres of the two clades, see Bolhuis et al 2010 doi:10.1038/nrn2931

• The second line of theoretical motivation behind the study is the Social Complexity Hypothesis for Communicative Complexity. There are some points I would like to raise here as well. For example, in line 17: I am rather confused by the reasoning behind the statement “Although supported in specific phylogenetic lineages, evidence for the SCHCC is often inconsistent” then go on to argue that it “is drawn largely from a small number of well-studied taxa, and frequently fails to consider a species’ full vocal repertoire”. If the hypothesis is inconsistent, do the authors think it is because it relies on limited taxa and few of their calls only? The criticism of the hypothesis is largely based on the general use of the term “social complexity” without standardised metric. Leighton and Birmingham (2021), and Leighton (2017) attempted to address this critique by correlating vocal complexity, measured as functional repertoire size, to specific aspects of social complexity, like group size, group stability, length of social bond etc. They found that cooperative breeding in particular explained this measure of vocal complexity, but was not the only factor behind it, but rather that ecological factors like foraging strata, contributed to explaining it.

I think therefore that the authors might want to consider that, since both the study species and the closely related Griffon vulture used for comparison are colonial breeders, this aspect of sociality could be the driver of social complexity in the species.

• How did the authors ascertain the captive conditions allowed for the production of the full repertoire? When captivity limits the full gamut of social contexts, particularly those that involve movement? Maybe emphasise this restriction of full natural behaviour imposed by captivity in the introduction and methods, because the introduction (Lines 211 – 213) currently argues for the importance of using the full vocal repertoire to study the evolution of a species communication system. Same applies for lines 326-329, I do not think it is accurate to state these contexts will result in the full gamut of the repertoire, seeing that they are captive and their main behaviour of movement via flying is not part of the study.

• Line 27- 28 “The Rüppell’s vulture repertoire exceeds the average repertoire size

observed across birds and is likely an adaptation to this species’ social life”, and Line 197 – 198 “12 distinct calls make it one of the largest repertoire sizes in birds” Maybe it is worth emphasising that you are talking here about non passerines, because song repertoires are on average much larger, particularly those of mimics. The Leighton and Birmingham, 2021 paper cited by the authors uses functional repertoire sizes, not absolute repertoire sizes, and reports only calls, not song types. the 12 – 16 call types found in this study are below average according to figure 1 of the cited paper.

• Method selection, how does the fuzzy k-means algorithm account for individual variation within the calls and the non independence of calls drawn from the same individual? when more than one call group together into a distinct cluster, how much variation is a mark of two different but graded call types of the same category, and what is the variation cutoff that signals the same call with individual variation among callers? For example, the chick and juvenile begging calls, the difference can either be the effect of age and development, but could also be the result of individual differences that persisted past developmental stages. In the Chen and Weins (2020) reference, it is stated that the within call type variation can be accounted for by the caller’s affective state, but there is a large body of literature that account for the within call variation by individual differences, either those that reflect morphological differences or markers of group membership among others. It is necessary to clarify this point in the introduction.

• Similar to the previous point, when considering hisses and whistle hisses, bray and bray hisses, MG and MG screechs, why assume they are different calls when it has a hiss or a screech component, and not just a syntactic combination of the call and a hiss or a call and screech?

• Finally, regarding the data availability, the authors stated that there are no restrictions on the data and that it is all readily available, but I found no link to the dataset or the R pipeline code. It is a good idea to include those along with the provided call exemplars for full transparency.

Line specific comments

• 279 – 285 – what about the prehatching parent calls? Do they vocalise to the unhatched eggs?

• Line 287 – 297 at what distance were these vocalisations recorded? And how did the experimenter decide at the appropriate distance given the various calls with different audible ranges?

• 288-293 are hyenas and snakes competitors only or predators? What are the predators of the vultures, particularly at the nest?

• Line 336 – 338 – what was the cutoff for the SNR?

• Figure 2 C colours are very faint for some calls, consider modifying the plot to maybe add border to the dots or replace the very light colours with darker shades.

• Line 425: “ In our analysis, we generated a single UMAP using the ‘umap’

function from the ‘uwot’ R package, and coloured the datapoints based on the subjective call labels determined by an observer”. Can you clarify the “subjective” nature of this labelling? It is relevant to how the reader will interpret figure 2.

• Following from the previous point: The call types in Figure 2, panel A and their names were not introduced prior to this point nor matched to the contexts in table 1. The first mention of call types was in line 351 stating the authors followed Romani et al 2002, but it did not mention the number of call types nor their names and contexts. it should be made clearer how good of a fit the observed calls in this study matched the cited study, and best to introduce the calls in table 1, matching them to context.

• Did the colouring of the datapoints in figure 2 panel A use the calls listed in Romani et al as a reference? If so, why did you call it “subjective” labelling, a descriptor that suggests the observer classified the different call types visually or audibly. Figure 3 introduces the call types and the matching contexts; it would be clearer if this is introduced earlier for the manual matching.

• Line 464-465 table S7 could not be found in the manuscript, I am guessing it provides the matched call types and contexts resulting from the Pearson’s X2 test, but the “subjective” matching should have been introduced first, to drive home the point of this analysis

• Figure 3 and the X2 test results: the test assumes the calls to be independent samples, but I am assuming many of the calls came from the same individual or calling bout? If that is true then the effective sample size is smaller than the stated number of calls (pseudo replication), which will result in an inflated value of the test and its significance. The test also assumes sufficient expected counts for each cell, which is not the case as seen in the sparsity of some call types in figure 3. Perhaps using a linear modelling approach that controls for repeated measures would be more appropriate, and potentially collapsing some of the rare call categories into those that are acoustically and contextually similar.

• Line 566 typicality threshold. If the typicality coefficient Line 413 – 417 is below the lower typicality threshold, why did the authors consider it graded call? Is it because of the consistent contexts?

• Line 580 table S8 cannot be found in the manuscript

• Line 644 – 650, the opening sentence is not very informative here. Referring back to my first two points, maybe stating that hypothesis is contested without clarification of how your study addresses that is not a clear motivation for conducting the study.

• Lines 652 – 653 : a common ancestor is not the only mechanism by which related species can share an adaptation, and since the study does not look into how the vocal repertoire evolved, it is not necessary to make a statement about it.

• Lines 661 -663 : as mentioned above, the figure in Leighton and Birmingham does not support this statement.

• 681 – 683 – what are the spectral characteristics in question? And how do the authors link those to the function of the three call types?

• Lines 763 – 771 : did the authors explore the existence of nest alarm calling? Despite not having natural predators as adults, the young are still vulnerable at the nest, and it is an interesting point to mention if it has been researched.

**********

what does this mean?). If published, this will include your full peer review and any attached files.

If you choose “no”, your identity will remain anonymous but your review may still be made public.

**Do you want your identity to be public for this peer review?**  For information about this choice, including consent withdrawal, please see our Privacy Policy

Reviewer #1: **Yes:**  Dina A. A. Mostafa

**********

[NOTE: If reviewer comments were submitted as an attachment file, they will be attached to this email and accessible via the submission site. Please log into your account, locate the manuscript record, and check for the action link "View Attachments". If this link does not appear, there are no attachment files.]

To ensure your figures meet our technical requirements, please review our figure guidelines: https://journals.plos.org/plosone/s/figures

You may also use PLOS’s free figure tool, NAAS, to help you prepare publication quality figures: https://journals.plos.org/plosone/s/figures#loc-tools-for-figure-preparation.

NAAS will assess whether your figures meet our technical requirements by comparing each figure against our figure specifications.

Attachment Submitted filename: renamed_41bea.docx

# Author comment: Author response to Decision Letter 1

**DOI:** 10.1371/journal.pone.0360246.r002

2 Sep 2026

[Note: This is also provided in a word document alongside other files]

Dear Dr. Brenton Cooper,

We sincerely appreciate the time and effort you and the reviewer have dedicated to evaluating our manuscript titled "The complex, graded vocal repertoire of the social Rüppell’s vulture”. We appreciate the opportunity to revise our manuscript based on the reviewer’s feedback, which has helped us improve the clarity and strength of our study and hereby resubmit a revised draft for your consideration.

Below, we provide detailed responses to each point raised during the review process. For clarity and ease of reference, we have reproduced the comments from the decision letter and included our responses directly beneath each one in blue. To facilitate navigation, all editor and reviewer comments have been numbered. As requested, all revisions have been made using track changes, and the corresponding line numbers in the revised manuscript (“Revised Rüppell’s vulture manuscript”) are indicated in red below; all changes are also visible in the tracked-changes copy.

Comments and Responses

Editor

Comment 1: First, in aves neuronal density, rather than size is correlated with sophisticated behaviors. This point requires careful clarification.

Response:

Thank you for this comment. We agree that brain size is a poor proxy for cognitive complexity in many taxa, which is partly why this study moves away from broad measures of encephalisation towards more specific behavioural domains. In response, we have clarified this point in the first paragraph of the Introduction and explicitly highlight that differences in neural architecture limit the generalisability of the social brain hypothesis across taxa.

L30-33: “Moreover, differences in neural architecture further complicate its application beyond mammals, particularly in birds, where neuronal density is often a stronger predictor of cognitive performance than brain size (Olkowicz et al., 2016), and in insects, which achieve remarkable behavioural complexity despite having relatively few neurons (Giurfa, 2013).”

Editor

Comment 2: Second, to accurately analyze the quality of the statistical analyses, please include source code for your analyses conducted in R and links to the data repository for your analyses. Additional data requested by the reviewer can be included in supplementary or additional figures.

Response:

Thank you for this suggestion. We have created a Zenodo data repository containing the raw data and R code required to reproduce our analyses. The repository will be publicly released upon publication of the manuscript under DOI: 10.5281/zenodo.20928811. For the purposes of peer review, it is currently accessible via the following restricted link:

https://zenodo.org/records/20928812?preview=1&token=eyJhbGciOiJIUzUxMiIsImlhdCI6MTc4NDQ2Njg3NSwiZXhwIjoxNzkzNDA0Nzk5fQ.eyJpZCI6IjNiNjU4OThkLWU5NzMtNDg4OC1hNDJjLWQxNGI3YmY1OTIzNSIsImRhdGEiOnt9LCJyYW5kb20iOiJlNjM3NTkxM2M0ZmM2ZWYzYTE3ZDZlMzNlNTkzZDBjNCJ9.J311ZZSGdhZCDR1S4InMt6bj-PYuktQptcW3dAs_vsiey9mIhH8k64LTL_BMvv6QA7C4oStPpVko9cREqhUBug

Reviewer:

Comment 1: My main questions deal with the theoretical motivation for this study. The authors cite the literature correlating relative brain size to increased cognitive functions, and propose a link to vocal complexity. Increased encephalisation has only been linked to higher brain functions in primates as the authors pointed out, but was found to be a poor measure of the same cognitive abilities in Aves, because the neural architecture is different when comparing the vocal learning centres of the two clades, see Bolhuis et al 2010 doi:10.1038/nrn2931

Response:

We thank the reviewer for this helpful comment. We agree that relative brain size is a less reliable proxy for cognitive performance in birds than in mammals, due to differences in neural architecture, including the organisation of specialised brain regions and the importance of neuronal density.

This point aligns with our original intention in the introduction, which was to briefly introduce the social brain hypothesis and associated use of encephalisation, while also motivating why this proxy is limited, particularly in non-mammalian taxa. This sets up the rationale for shifting focus away from broad measures of brain size towards more specific and functionally grounded aspects of social cognition, such as communication.

In the revised manuscript, we have clarified this transition more explicitly and reduced emphasis on encephalisation and focus more on communication, which is more directly relevant to the aims of this study.

L30-33: “Moreover, differences in neural architecture further complicate its application beyond mammals, particularly in birds, where neuronal density is often a stronger predictor of cognitive performance than brain size (Olkowicz et al., 2016), and insects, which achieve remarkable behavioural complexity despite having relatively few neurons (Giurfa, 2013).”

Comment 2: The second line of theoretical motivation behind the study is the Social Complexity Hypothesis for Communicative Complexity. There are some points I would like to raise here as well. For example, in line 17: I am rather confused by the reasoning behind the statement “Although supported in specific phylogenetic lineages, evidence for the SCHCC is often inconsistent” then go on to argue that it “is drawn largely from a small number of well-studied taxa, and frequently fails to consider a species’ full vocal repertoire”. If the hypothesis is inconsistent, do the authors think it is because it relies on limited taxa and few of their calls only? The criticism of the hypothesis is largely based on the general use of the term “social complexity” without standardised metric. Leighton and Birmingham (2021), and Leighton (2017) attempted to address this critique by correlating vocal complexity, measured as functional repertoire size, to specific aspects of social complexity, like group size, group stability, length of social bond etc. They found that cooperative breeding in particular explained this measure of vocal complexity, but was not the only factor behind it, but rather that ecological factors like foraging strata, contributed to explaining it.

I think therefore that the authors might want to consider that, since both the study species and the closely related Griffon vulture used for comparison are colonial breeders, this aspect of sociality could be the driver of social complexity in the species.

Response:

We thank the reviewer for this comment. We agree that our original wording did not clearly distinguish between limitations of the current evidence base and limitations of the SCHCC itself. In response, we have substantially revised the introduction to clarify that the SCHCC remains an important and influential framework, but that support for its predictions remains mixed across taxa and studies. We now also identify more clearly the barriers currently impacting its generalizability as an evolutionary framework.

Specifically, our intention was not to imply that the hypothesis is unsupported simply because it has been tested in a limited number of taxa or because studies often examine only part of a species’ vocal repertoire. Rather, we wished to highlight several factors that currently limit broad conclusions, including variation in how both social and communicative complexity are defined and measured, methodological differences among studies that complicate direct comparisons and meta-analyses (e.g., Leighton & Birmingham, 2021; McComb & Semple, 2005), a strong reliance on a relatively small number of model taxa to draw broad conclusions for all animals, and growing evidence that ecological, environmental, and phylogenetic factors may also play important roles in shaping communicative complexity.

We have therefore revised the introduction to better reflect the current state of the field and the reasons why additional tests of the SCHCC in underexplored taxonomic groups remain important, which is the motivation for our study that introduces vultures as a well-suited guild for such tests. See paragraph starting on Line 63.

Additionally, we have revised the sentence the reviewer highlighted in the abstract on what was line 17 to clarify the motivation further. L6-9: “Although several studies provide support for the SCHCC, its empirical basis remains limited and inconsistent, as evidence is drawn predominantly from a small number of well-studied taxa (primarily primates), often neglects species’ complete vocal repertoires, and employs inconsistent approaches for assessing social and vocal complexity.”

Comment 3: How did the authors ascertain the captive conditions allowed for the production of the full repertoire? When captivity limits the full gamut of social contexts, particularly those that involve movement? Maybe emphasise this restriction of full natural behaviour imposed by captivity in the introduction and methods, because the introduction (Lines 211 – 213) currently argues for the importance of using the full vocal repertoire to study the evolution of a species communication system. Same applies for lines 326-329, I do not think it is accurate to state these contexts will result in the full gamut of the repertoire, seeing that they are captive and their main behaviour of movement via flying is not part of the study.

Response:

We thank the reviewer for raising this important point. We agree that captivity does not allow access to the full range of natural behavioural contexts, particularly those associated with long-distance movement and soaring flight, and therefore cannot guarantee that the entire vocal repertoire is expressed under the conditions studied. We have clarified this limitation in the Discussion. Nevertheless, through 150 hours of observations in many contexts, we made every effort to sample as many call types as we can in captivity.

We also now explicitly acknowledge that additional context-dependent vocalisations, particularly those potentially associated with in-flight social interactions and movement coordination, may occur in wild populations and were not accessible in this study.

L790-799: “We note, however, that our study was conducted in captivity, which likely limits the expression of certain behavioural contexts and, consequently, the production of associated vocalisations. It is therefore possible that additional call types occur in wild populations, meaning the repertoire described here may represent a conservative estimate of the species' full vocal repertoire. For Gyps vultures, one important context absent from captivity is the movement interactions that occur during soaring flight. Here, they routinely travel in groups and rely on aerial information networks when making foraging decisions while soaring at high altitudes (Cortés-Avizanda et al., 2014), creating opportunities for social interactions that do not occur in captive settings. If vocal communication contributes to coordinating these movements or maintaining group cohesion during flight, additional context-specific vocalisations may exist that were not captured in the present study.”

Comment 4: Line 27- 28 “The Rüppell’s vulture repertoire exceeds the average repertoire size observed across birds and is likely an adaptation to this species’ social life”, and Line 197 – 198 “12 distinct calls make it one of the largest repertoire sizes in birds” Maybe it is worth emphasising that you are talking here about non passerines, because song repertoires are on average much larger, particularly those of mimics. The Leighton and Birmingham, 2021 paper cited by the authors uses functional repertoire sizes, not absolute repertoire sizes, and reports only calls, not song types. the 12 – 16 call types found in this study are below average according to figure 1 of the cited paper.

Response:

We thank the reviewer for raising this important point and for allowing us to clarify our arguments. We are indeed talking about calls sounds associated with distinct behavioral contexts, not song, so functional repertoire size, but are not specifically comparing with non-passerines only, as from passerines one can also create a functional repertoire size, as Leighton and Birmingham in fact do. That said, we are a bit confused by the comment about figure 1, as that figure in fact does not show any average, but only the spread. We acknowledge that there are birds with larger repertoire sizes and have therefore now changed our wording in (what was previously) line 197-198, to now read “larger” rather than “largest”. Nevertheless, we maintain our conclusion that they exceed the average, since that conclusion stems for a comparison to figure 2 of said paper, where they do show median averages (of both cooperative or non-cooperative breeders, as well as from species with high, moderate, or minimal cohesiveness) that all do not exceed 10, and 12-16 is thus higher.

We note that their synthesis is based on functional repertoire sizes derived from calls across taxa and therefore provides a relevant comparative framework for interpreting call-based repertoires. We have also expanded the manuscript text to better contextualise these findings within non-passerine and colonial species, while avoiding over-interpretation of cross-study quantitative comparisons.

L19: We add “call” to specify here that we are talking about call repertoire, not song repertoire.

L638-643: “The range of call types documented in this study indicates that Rüppell’s vultures possess a highly complex vocal repertoire, on par or exceeding that of the griffon vulture (Romani et al., 2022). This therefore places the Rüppell’s vulture vocal repertoire well above the average call diversity among birds (Leighton & Birmingham, 2021), being comparable to several highly social primates (Fichtel & Kappeler, 2022; Fischer & Hammerschmidt, 2002; Grimm, 1967; Hohmann, 1991; Palombit, 1992), and significantly more complex than those of many other colony-breeding non-passerines (Favaro et al., 2014; Mäkelin et al., 2021; Osiecka et al., 2023), and raptors (Huang et al., 2021).”

Comment 5: Method selection, how does the fuzzy k-means algorithm account for individual variation within the calls and the non independence of calls drawn from the same individual? when more than one call group together into a distinct cluster, how much variation is a mark of two different but graded call types of the same category, and what is the variation cutoff that signals the same call with individual variation among callers? For example, the chick and juvenile begging calls, the difference can either be the effect of age and development, but could also be the result of individual differences that persisted past developmental stages. In the Chen and Weins (2020) reference, it is stated that the within call type variation can be accounted for by the caller’s affective state, but there is a large body of literature that account for the within call variation by individual differences, either those that reflect morphological differences or markers of group membership among others. It is necessary to clarify this point in the introduction.

Response:

We thank the reviewer for this important and conceptually detailed comment regarding the interpretation of the fuzzy clustering approach and the sources of within-call variation. The fuzzy clustering analysis used here is an unsupervised method that groups observations solely based on similarity in multidimensional acoustic feature space. It does not incorporate individual identity, age class, or behavioural context as input variables, and therefore does not explicitly partition variance attributable to these factors. Consequently, sources of variation such as individual differences, developmental stage, and affective state are not modelled separately, but all influence the acoustic feature space from which clustering is derived. Importantly, because the output produces continuous membership values that reflect relative similarity to cluster centroids, clusters should be interpreted as regions of acoustic similarity structure rather than discrete categories, and within-cluster variation should be understood as a composite of all underlying sources of acoustic variability (e.g., individuality, emotion, demography, morphology, noise) present in the data.

We agree with the reviewer that within-call variation may arise from multiple biological mechanisms, including individual identity, ontogeny, and internal state, as well as from graded signal structure. However, these components cannot be disentangled with the present analysis and would require individually identified longitudinal recordings, which are beyond the scope of this descriptive study.

With respect to the concern about chick and juvenile begging calls, we clarify that both were assigned to the same acoustic cluster by the fuzzy clustering analysis. Any distinction reported in the manuscript refers to behavioural context (developmental stage) rather than separation into distinct acoustic clusters (see Fig 2.B.). We clarify this in the Results section to avoid ambiguity in interpretation:

L425-431: “Note, because the output produces continuous membership values that reflect relative similarity to cluster centroids, clusters should be interpreted as regions of acoustic similarity in a continuous feature space rather than discrete categories. Accordingly, within-call variation reflects the combined influence of all sources of acoustic variation present in the data, which may include but are not limited to individual differences, developmental stage, affective state, morphological constraints, and recording noise, although these sources cannot be disentangled within the present analytical framework.”

Comment 6: Similar to the previous point, when considering hisses and whistle hisses, bray and bray hisses, MG and MG screechs, why assume they are different calls when it has a hiss or a screech component, and not just a syntactic combination of the call and a hiss or a call and screech?

Response:

We thank the reviewer for raising this interesting point regarding the classification of structurally similar vocalisations. This issue highlights a broader challenge in working with highly graded vocal repertoires, where call types often exist along a continuum of acoustic variation and clear boundaries between categories are inherently difficult to define (as stated on line 655-656).

Here, call type assignments were based on descriptions in Romani et al. (2022), combined with our own systematic evaluation of audio-visual structure. However, we agree that in several cases (e.g. bray vs. bray hisses and MG vs. MG screeches), acoustic features suggest partial overlap between categories, and these should be interpreted as graded variants rather than strictly discrete call types (see line 543, and lines 595-596). The hiss and whistle-hiss do not represent syntactic variants, however, and differ in their appearance and sound. This is further supported by the median typicality coefficients, which found that the MGs and MG-screeches had a highly graded structure, suggesting that they may are highly flexible (likely a result of their combinatorial structure).

With respect to the suggestion that these represent syntactic combinations, we note that demonstrating syntactic structure requires evidence of stable combinatorial rules governing the sequencing and/or functional modification of constituent elements. The present study does not test such structural rules. We therefore refer to these vocalisations more cautiously as potentially combinatorial or compound calls, in which acoustic elements co-occur, without implying syntactic organisation.

Finally, we have clarified in the manuscript that the Rüppell’s vulture repertoire exhibits substantial gradation, and that for some call types, boundaries are descriptive rather than absolute, reflecting continuity in acoustic space rather than discrete categorical separation.

L425-427: “Note, because the output produces continuous membership values that reflect relative similarity to cluster centroids, clusters should be interpreted as regions of acoustic similarity in a continuous feature space rather than discrete categories”

Comment 7: Finally, regarding the data availability, the authors stated that there are no restrictions on the data and that it is all readily available, but I found no link to the dataset or the R pipeline code. It is a good idea to include those along with the provided call exemplars for full transparency.

Response:

Thank you for raising this point. We included the full raw data set used in our fuzzy clustering analysis as supplemental table S1, however agree that including it in a repository will improve the transparency further. In response, we have now created a repository in Zenodo which includes our dataset and R code under the DOI 10.5281/zenodo.20928811. Supplemental materials still include the additional call exemplars, videos, and supplemental tables.

For peer review purposes, the repository currently has restricted access however can be viewed through this link:

https://zenodo.org/records/20928812?preview=1&token=eyJhbGciOiJIUzUxMiIsImlhdCI6MTc4NDQ2Njg3NSwiZXhwIjoxNzkzNDA0Nzk5fQ.eyJpZCI6IjNiNjU4OThkLWU5NzMtNDg4OC1hNDJjLWQxNGI3YmY1OTIzNSIsImRhdGEiOnt9LCJyYW5kb20iOiJlNjM3NTkxM2M0ZmM2ZWYzYTE3ZDZlMzNlNTkzZDBjNCJ9.J311ZZSGdhZCDR1S4InMt6bj-PYuktQptcW3dAs_vsiey9mIhH8k64LTL_BMvv6QA7C4oStPpVko9cREqhUBug

Comment 8: 279 – 285 – what about the prehatching parent calls? Do they vocalise to the unhatched eggs?

Response:

We thank the reviewer for bringing up this point and we would like to clarify that we did not detect any parent calls to the unhatched egg. We monitored the nests using microphones when they were incubating model eggs. We would like to note that the parents never heard the prehatching chick vocalisations because of the husbandry procedure and therefore do not know how if that elicits parent calls. We clarify this point in the manuscript.

L468-469: “Given the husbandry procedure of artificial incubation, we could not determine whether parents also vocalise back to the chick in the egg in response to egg calls.”

Comment 9: Line 287 – 297 at what distance were these vocalisations recorded? And how did the experimenter decide at the appropriate distance given the various calls with different audible ranges?

Response:

We thank the reviewer for highlighting this point. We have clarified the recording distances in the Methods. Recordings in the general group and feeding contexts were made from approximately 2–10 m of the focal vulture. We did not apply a fixed distance threshold for different call types. Instead, each recording was assessed on a case-by-case basis based on the quality of the acoustic signal using audio-visual inspection of the spectrogram. This approach was necessary because audio quality depends not only on the call type itself but also on environmental conditions such as weather, background noise from zoo visitors, and the position and orientation of the calling bird (see the "Acoustic and Behaviour Analysis" section for details of the selection criteria) which affect how calls attenuate over distance.

L275-276: “These recordings were made at between approximately two and ten meters of the calling individual.”

Comment 10: 288-293 are hyenas and snakes competitors only or predators? What are the predators of the vultures, particularly at the nest?

Response:

Thank you for this comment. To clarify, hyenas are mammalian competitors for food, while snakes are a possible threat (particularly at nests). We clarify this in the manuscript. Generally, however, because this species nests in very high, steep cliffs, there are few predators that threaten chicks and eggs. Some potential predators include other raptors (e.g., black eagles), corvids, and more rarely baboons. In the aviary at Rotterdam Zoo, there are black kites (no eagles), and at Vogelpark Avifauna there were pied crows. We did not detect any calls in response to the presence of these species near their nests.

In response, we have added details in the methods to clarify the snake represents a potential threat, while we also clarify in our discussion what kinds of predators they face at nests.

L260: “simulating a potential threat to the colony”

L742-745: “Furthermore, although some potential nest predators exist (e.g., large raptors and corvids), the species’ steep cliff nesting sites are largely inaccessible to most terrestrial predators, substantially reducing predation pressure on eggs and chicks.”

Comment 11: Line 336 – 338 – what was the cutoff for the SNR?

Response:

We thank the reviewer for this comment. We did not apply a fixed signal-to-noise ratio threshold when selecting calls for analysis. Instead, recordings were assessed on a case-by-case basis according to the quality criteria described in the Methods. Specifically, calls were retained only when they were sufficiently clear for reliable annotation and acoustic measurement, with start and end times and minimum and maximum frequencies unambiguously identifiable, without acute noise impacting measures. Because recording conditions varied considerably across contexts and call types vary in their acoustic energy, we considered this qualitative assessment to be more appropriate than imposing an arbitrary SNR cutoff. We clarify this in the Methods.

L306-308: “We further analysed only the calls that produced visibly clear spectrograms with a high signal-to-noise ratio without interference from background noises, such as other animals or weather, allowing us to reliably annotate calls and extract the parameters for analysis.”

Comment 12: Figure 2 C colours are very faint for some calls, consider modifying the plot to maybe add border to the dots or replace the very light colours with darker shades.

Response:

Thank you for bringing this to our attention. We agree that some of the colours used were quite faint in the original version of this figure and have taken this opportunity to improve the accessibility of all our figures. As such, the colour palette in Fig 2 has been adjusted, while we have adjusted figure 3 and figure 9’s colour palettes to keep them consistent with the new style.

See Figure 2.

Comment 13: Line 425: “ In our analysis, we generated a single UMAP using the ‘umap’ function from the ‘uwot’ R package, and coloured the datapoints based on the subjective call labels determined by an observer”. Can you clarify the “subjective” nature of this labelling? It is relevant to how the reader will interpret figure 2.

Response:

We thank the reviewer for highlighting this point and agree that the term “subjective” may have been misleading in this context. The call labels were not assigned arbitrarily but were based on systematic audio-visual inspection of each recording (i.e., simultaneous examination of spectrograms and playback of calls). Initial call-type categories were informed by those described in Romani et al. (2022) for griffon vultures and were subsequently refined and expanded during extensive data collection and field experience with Rüppell’s vultures by C.P. As such, these classifications represent knowledge-based annotations. We have revised the manuscript to replace “subjective call labels” with “knowledge-based call labels” to better reflect this procedure.

L321-324: Additionally, each call given knowledge-based call type labels based on visual and acoustic features. The initial call-type scheme followed the griffon vulture repertoire described in Romani et al. (2022) and was subsequently refined and updated by C.P. during extensive observations of Rüppell’s vulture calls during data collection.

Comment 14: Following from the previous point: The call types in Figure 2, panel A and their names were not introduced prior to this point nor matched to the contexts in table 1. The first mention of call types was in line 351 stating the authors followed Romani et al 2002, but it did not mention the number of call types nor their names and contexts. it should be made clearer how good of a fit the observed calls in this study matched the cited study, and best to introduce the calls in table 1, matching them to context.

Response:

We thank the reviewer for this comment. As addressed in our response to Comment 13, we have clarified the process used to assign call labels (Lines 431–434). In addition, we now introduce the call types by name in the Methods section, specifying which correspond to those previously described by Romani et al. (2022) in griffon vultures and which are described here for the first time. We believe these revisions make it clearer how closely the observed Rüppell's vulture call repertoire corresponds to that described in the cited study.

We have not added call type-context associations to Table 1, as these relationships represent findings from the present study rather than criteria used for call classification. Instead, we present the association between call types and behavioural contexts in the Results section in Figure 3, where these relationships are formally presented.

L324-329: Many call types previously described in griffon vultures were also identified in our dataset, including the hiss, bray, bray-hiss, grunt, groan, metallic groan, and small groan. The chicken coo, chicken groan, juvenile scream, and sharp scream were likewise documented in both species; however, we renamed these call types to chick call, chick begging call, juvenile begging call, and screech, respectively, to better reflect their acoustic properties and the behavioural contexts in which they are produced. We also described several new call types, including the sex groan, egg call, provision call, and whistle-hiss.

Comment 15: Did the colouring of the datapoints in figure 2 panel A use the calls listed in Romani et al as a reference? If so, why did you call it “subjective” labelling, a descriptor that suggests the observer classified the different call types visually or audibly. Figure 3 introduces the call types and the matching contexts; it would be clearer if this is introduced earlier for the manual matching.

Response:

Thank you for this comment. As partly addressed in our responses to Comments 13 and 14, we have revised our terminology and now refer to these as knowledge-based call type classifications rather than subjective labels.

We have clarified the call types and their basis for classification earlier in the manuscript (see lines 324-329); however, we have retained the current order of presentation. The aim of this study was first to quantitatively and systematically characterise the structure of the Rüppell’s vulture vocal repertoire and identify call types, and subsequently to examine how these call types are associated with behavioural contexts. We therefore consider the contextual associations presented later in the Results to be an interpretation of the identified call types rather than information used to define them

Comment 16: Line 464-465 table S7 could not be found in the manuscript, I am guessing it provides the matched call types and contexts resulting from the Pearson’s X2 test, but the “subjective” matching should have been introduced first, to drive home the point of this analysis

Response:

We thank the reviewer for their comment and for the opportunity to clarify this point. Table S7 (along with all other supplementary tables) is included in the supplementary materials ZIP folder submitted with the original manuscript. We apologise if its location was not sufficiently clear in the current version of the submission. Table S7 indeed presents the distribution of behavioural contexts associated with each call type, as used in the Pearson’s χ² analysis. We also take note of the reviewer’s suggestion regarding terminology and presentation and have revised the manuscript to ensure that the description of the call classification procedure is introduced earlier.

Comment 17: Figure 3 and the X2 test results: the test assumes the calls to be independent samples, but I am assuming many of the calls came from the same individual or calling bout? If that is true then the effective sample size is smaller than the stated number of calls (pseudo replication), which will result in an inflated value of the test and its significance. The test also assumes sufficient expected counts for each cell, which is not the case as seen in the sparsity of some call types in figure 3. Perhaps using a linear modelling approach that controls for repeated measures would be more appropriate, and potentially collapsing some of the rare call categories into those that are acoustically and contextually similar.

Response:

We thank the reviewer for this important comment regarding the assumptions of the χ² test. We agree that the test assumes independence of observations, and that multiple calls may originate from the same individuals, which could introduce non-independence and reduce the effective sample size.

In response to this comment, we now explicitly clarify that the χ² analysis is used in an exploratory framework to assess whether call types and behavioural contexts are non-randomly associated at the population level. The aim of this analysis is therefore to describe overall structure in the observed dataset rather than to make fine-scale inferential claims, and no post-hoc comparisons are conducted.

A mixed-effects modelling approach was considered; however, this was not feasible for the present dataset. Individual identity could not be reliably assigned for a substantial proportion of calls, particularly during feeding interactions where multiple birds were vocalising simultaneously and callers could not always be unambiguously identified. In addition, due to the opportunistic nature of the recordings, individuals are not consistently identifiable. While we acknowledge potential non-independence within calling bouts, there is no consistent or widely accepted definition of a “bout” across the behavioural contexts included here, making bout-level structuring impractical.

Regarding the reviewer's concern about low expected cell counts arising from rare call types, the χ² test was performed using Monte Carlo simulated p-values (10,000 replicates). We selected this approach because it avoids reliance on the asymptotic χ² distribution and is recommended for sparse contingency tables with low expected cell counts. We therefore do not consider it necessary to collapse the rarer call categories, as the simulated p-value approach is robust to sparse contingency tables and preserving these categories better reflects the observed vocal repertoire.

We note that the same analytical approach has previously been used in Romani et al. (2022) to test for associations between call type and behavioural context, supporting its use as an exploratory method for evaluating population-level structure in call usage. We therefore retain the χ² analysis under this interpretation. As shown in Figure 3, the data exhibit a clear pattern of non-random association between call types and behavioural contexts.

L444-447: “We also found a clear and strong association between call types and the contexts in which they are produced (χ² = 15867, p < 0.0001; Figure 3 and Table S7). Because multiple calls originate from the same individuals or calling bouts, the associated p-value should be interpreted cautiously. Nevertheless, the observed distribution reveals a clear, non-random structuring of call use across behavioural contexts.”

Comment 18: Line 566 typicality threshold. If the typicality coefficient Line 413 – 417 is below the lower typicality threshold, why did the authors consider it graded call? Is it because of the consistent contexts?

Response:

We thank the reviewer for this question, which allows us to clarify the classification criteria. As defined in Wadewitz et al. (2015), call types are categorised based on their median typicality coefficient relative to two thresholds: call types with median typicality coefficients above the upper (typicality) threshold are considered discrete, those below the lower (atypicality) threshold are considered graded, and those falling between the two thresholds are classified as intermediate.

Accordingly, when a call type has a median typicality coefficient below the lower threshold, it is classified as graded, irrespective of its contextual consistency. This classification is therefore based solely on the position of the median typicality coefficient relative to the predefined thresholds, not on context.

Although correct, the wording used on what was line 566 was indeed confusing, and we have clarified this in the manuscript to improve readability.

L545-547: “97.4% of the 727 metallic groans had typicality coefficients below the typicality threshold (median TC = 0.14, IQR = 0.14), indicating an extreme degree of acoustic variation and structural gradation (Figure 8).”

Comment 19: Line 580 table S8 cannot be found in the manuscript

Response:

We thank the reviewer for their comment. As with Table S7, Table S8 was included in the supplementary materials ZIP folder submitted with the original manuscript and is available there in full. We apologise if its location was not sufficiently clear in the current version of the submission.

Comment 20: Line 644 – 650, the opening sentence is not very informative here. Referring back to my first two points, maybe stating that hypothesis is contested without clarification of how your study addresses that is not a clear motivation for conducting the study.

Response:

We are grateful to the reviewer for pointing this out. In response, we have rewritten the first paragraph of our discussion to clarify the motivation of our present study, while we have also further explained this in our introduction to highlight the reason we believe this work is important.

L620-625: “Social complexity has been proposed as an key evolutionary driver of communicative complexity (Freeberg et al., 2012; Peckre et al., 2019), yet empirical support remains mixed within a limited set of model species. Expanding the taxonomic breadth of species used to test hypotheses on the evolution of communication is therefore essential, although this first requires substantial foundational work in non-model systems. To facilitate this, we characterised the vocal repertoire of the socially complex Rüppell’s vulture (van Dijk et al., in prep.), building on previous work in griffon vultures (Romani et al., 2024).”

Comment 21: Lines 652 – 653 : a common ancestor is not the only mechanism by which related species can share an adaptation, and since the study does not look into how the vocal repertoire evolved, it is not necessary to make a statement about it.

Response:

Thank you for this comment. We agree that the present study does not directly investigate the evolutionary history of the vocal repertoire. Our intention was not to infer the mechanism by which the repertoire evolved, but rather to note that the substantial similarity between the repertoires of two closely related sister species is most parsimoniously explained by inheritance from a recent common ancestor. We recognise that alternative evolutionary scenarios cannot be excluded, however given the strong similarity it is difficult to argue that they can be the result of another evolutionary scenario. To avoid overstating this inference, we have revised the text to make clear that shared ancestry is presented as the most parsimonious interpretation, rather than a conclusion directly tested by this study.

L629-633: “Like griffon vultures, Rüppell’s vultures use a diverse array of vocalisations across a range of social contexts, with strong similarities in the repertoires of the two closely related species. This pattern is most parsimoniously explained by the hypothesis that the complex vocal system in these species likely evolved in a highly social common ancestor and has been retained in both lineages, reflecting the expectations of the SCHCC.”

Comment 22: Lines 661 -663 : as mentioned above, the figure in Leighton and Birmingham does not support this statement.

Response:

We thank the reviewer for this comment. We believe we have addressed this point under comment 4. However, to briefly reiterate, we stand by our conclusion that the call repertoire of Rüppell’s vultures is above average when compared to figure 2 in Leighton and Birmingham (2021). To further contextualise it, we have also compared our repertoire size to other taxonomic groups including primates and other colonial non-passerines.

We have made the following changes to this section to better reflect the literature:

L638-643: “The range of call types documented in this study indicates that Rüppell’s vultures possess a highly complex vocal repertoire, on par or exceeding that of the griffon vulture (Romani et al., 2022). This therefore places the Rüppell’s vulture vocal repertoire well above the average call diversity among birds (Leighton & Birmingham, 2021), being comparable to several highly social primates (Fichtel & Kappeler, 2022; Fischer & Hammerschmidt, 2002; Grimm, 1967; Hohmann, 1991; Palombit, 1992), and significantly more complex than those of many other colony-nesting non-passerines (Favaro et al., 2014; Mäkelin et al., 2021; Osiecka et al., 2023), and raptors (Huang et al., 2021).”

Comment 23: 681 – 683 – what are the spectral characteristics in question? And how do the authors link those to the function of the three call types?

Response:

Thank you for this comment. We did not directly test which spectral parameters were similar, however rather make this speculation based on the overall resemblance of the spectrograms and sounds of these calls. We have amended this statement:

L660-662: “Begging calls are also acoustically similar to the screeches and brays produced during trophic competition (both being harsh, broadband calls; see Figures 4, 7, and 10), suggesting a potential developmental link between these vocal signals and food-related contexts”

Comment 24: Lines 763 – 771 : did the authors explore the existence of nest alarm calling? Despite not having natural predators as adults, the young are still vulnerable at the nest, and it is an interesting point to mention if it has been researched.

Response:

We thank the reviewer for this interesting suggestion. We did not explicitly test for the presence of chick alarm calls in this study, and therefore cannot draw definitive conclusions regarding this behaviour. However, it is worth noting that the aviary in which nest recordings were collected is shared with other bird of prey species, including black kites and marabou storks, providing potential exposure to overhead movement and disturbance. Within the available dataset, we did not observe any calls that could be clearly interpreted as alarm vocalisations in response to these species passing overhead. A dedicated experimental approach would be required to formally test this hypothesis, but such an approach falls outside the scope of the present study and would also raise ethical considerations regarding potential disturbance to vulnerable chicks. We have provided some more detail in the manuscript on this point

L206-210: “Alongside Rüppell’s vultures, other species in the aviaries included white-headed vultures (Trigonoceps occipitalis), white-backed vultures (Gyps africanus), hooded vultures (Necrosyrtes monachus), marabou storks (Leptoptilos crumenifer), black kites (Milvus migrans), pied crows (Corvus albus), southern ground hornbills (Bucorvus leadbeateri), and various smaller passerine and galliform species.”

L260-262: We also mention that these mixed species aviaries allowed us to look for potential calls in response to their co-housed species “Furthermore, since the vultures were housed with other large raptors, we could monitor their response to species which may pose a threat to their chicks (e.g., pied crows and black kites).”

Attachment Submitted filename: Response to Reviewers_PlosOne_19.07.26.docx

# Peer review: Decision Letter 1

**DOI:** 10.1371/journal.pone.0360246.r003

**Authors:** Brenton Cooper

15 Sep 2026

Dear Dr. Proudfoot,

Thank you for submitting your manuscript to PLOS One. After careful consideration, we feel that it has merit but does not fully meet PLOS One’s publication criteria as it currently stands. Therefore, we invite you to submit a revised version of the manuscript that addresses the points raised by the reviewer before a formal acceptance can be issued.

Please submit your revised manuscript by Oct 30 2026 11:59PM. If you will need more time than this to complete your revisions, please reply to this message or contact the journal office at plosone@plos.org. When you're ready to submit your revision, log on to https://www.editorialmanager.com/pone/ and select the 'Submissions Needing Revision' folder to locate your manuscript file.

- A letter that responds to each point raised by the academic editor and reviewer(s). You should upload this letter as a separate file labeled 'Response to Reviewers'.
- A marked-up copy of your manuscript that highlights changes made to the original version. You should upload this as a separate file labeled 'Revised Manuscript with Track Changes'.
- An unmarked version of your revised paper without tracked changes. You should upload this as a separate file labeled 'Manuscript'.

If you would like to make changes to your financial disclosure, please include your updated statement in your cover letter. Guidelines for resubmitting your figure files are available below the reviewer comments at the end of this letter.

If applicable, we recommend that you deposit your laboratory protocols in protocols.io to enhance the reproducibility of your results. Protocols.io assigns your protocol its own identifier (DOI) so that it can be cited independently in the future. For instructions see: https://journals.plos.org/plosone/s/submission-guidelines#loc-laboratory-protocols. Additionally, PLOS One offers an option for publishing peer-reviewed Lab Protocol articles, which describe protocols hosted on protocols.io. Read more information on sharing protocols at https://plos.org/protocols?utm_medium=editorial-email&utm_source=authorletters&utm_campaign=protocols.

As the corresponding author, your ORCID iD is verified in the submission system and will appear in the published article. PLOS supports the use of ORCID, and we encourage all coauthors to register for an ORCID iD and use it as well. Please encourage your coauthors to verify their ORCID iD within the submission system before final acceptance, as unverified ORCID iDs will not appear in the published article. *Only*  the individual author can complete the verification step; PLOS staff *cannot*  verify ORCID iDs on behalf of authors.

We look forward to receiving your revised manuscript.

Kind regards,

Brenton G. Cooper, Ph.D.

Academic Editor

PLOS One

Journal Requirements:

If the reviewer comments include a recommendation to cite specific previously published works, please review and evaluate these publications to determine whether they are relevant and should be cited. There is no requirement to cite these works unless the editor has indicated otherwise.

Please review your reference list to ensure that it is complete and correct. If you have cited papers that have been retracted, please include the rationale for doing so in the manuscript text, or remove these references and replace them with relevant current references. Any changes to the reference list should be mentioned in the rebuttal letter that accompanies your revised manuscript. If you need to cite a retracted article, indicate the article’s retracted status in the References list and also include a citation and full reference for the retraction notice.

[Note: HTML markup is below. Please do not edit.]

Reviewers' comments:

Reviewer's Responses to Questions

**Comments to the Author**

Reviewer #1: All comments have been addressed

**********

2. Is the manuscript technically sound, and do the data support the conclusions?

Reviewer #1: Yes

**********

3. Has the statistical analysis been performed appropriately and rigorously?

Reviewer #1: Yes

**********

4. Have the authors made all data underlying the findings in their manuscript fully available?

The PLOS Data policy

Reviewer #1: Yes

**********

5. Is the manuscript presented in an intelligible fashion and written in standard English?

Reviewer #1: Yes

**********

Reviewer #1: I have received the revised manuscript and reviewed the changes made, and I find the changes the authors made to effectively address the comments from the first round, resulting in a more replicable and accessible study. I do have minor follow up comments that I list below and hope the authors will find it useful.

General comment:

The introduction is very well written, concise and paints an interesting picture. I would however reorganise its parts to open with the link between social and communication complexity. The current first paragraph reads like it naturally follows from the second.

Specific comment:

• The X2 test results: The authors’ response regarding the low expected cell counts and how they relied on Monte Carlo simulated p-values sufficiently addresses my comment. My other concern regarding the non-independence of observations and how that violates the assumptions of the test, still stand. I appreciate that under the setup of data collection it was not possible to account for caller identity and that accordingly the analysis only serves an exploratory purpose. However, I do not agree that the current presentation of the results reflect this deviation from the test assumptions. The first half recommends the reader interpret the p-values cautiously, while the second half affirms the results to reveal “clear, non-random structuring of call use across behavioural contexts. This phrasing, along with the first two lines presenting the result assert a stronger claim than supported by the results of the X2 test, making the two statements presenting this result contradictory. The authors can still state the data descriptively show a strong distinction of call type production within context, without making an unsupported statement about statistical inference. The observed pattern of call production within context can be skewed to a higher contribution of a large number of calls belonging to one individual for example, without such pattern being detected by the test.

**********

what does this mean?). If published, this will include your full peer review and any attached files.

If you choose “no”, your identity will remain anonymous but your review may still be made public.

**Do you want your identity to be public for this peer review?**  For information about this choice, including consent withdrawal, please see our Privacy Policy

Reviewer #1: **Yes:**  Dina A. A. Mostafa

**********

[NOTE: If reviewer comments were submitted as an attachment file, they will be attached to this email and accessible via the submission site. Please log into your account, locate the manuscript record, and check for the action link "View Attachments". If this link does not appear, there are no attachment files.]

To ensure your figures meet our technical requirements, please review our figure guidelines: https://journals.plos.org/plosone/s/figures

You may also use PLOS’s free figure tool, NAAS, to help you prepare publication quality figures: https://journals.plos.org/plosone/s/figures#loc-tools-for-figure-preparation.

NAAS will assess whether your figures meet our technical requirements by comparing each figure against our figure specifications.

Attachment Submitted filename: renamed_77aa2.docx

# Author comment: Author response to Decision Letter 2

**DOI:** 10.1371/journal.pone.0360246.r004

22 Sep 2026

Dear Dr. Brenton Cooper,

We sincerely appreciate the time and effort you and the reviewer have dedicated to evaluating our manuscript titled "The complex, graded vocal repertoire of the social Rüppell’s vulture”. We appreciate the opportunity to revise our manuscript and address the reviewer’s two comments.

Below, we provide detailed responses to each point raised during the review process. For clarity and ease of reference, we have reproduced the comments from the decision letter and included our responses directly beneath each one in blue. To facilitate navigation, all editor and reviewer comments have been numbered. As requested, all revisions have been made using track changes, and the corresponding line numbers in the track changes revised manuscript are indicated in red below; all changes are also visible in the tracked-changes copy.

Comments and Responses

Reviewer 1

Comment 1: The introduction is very well written, concise and paints an interesting picture. I would however reorganise its parts to open with the link between social and communication complexity. The current first paragraph reads like it naturally follows from the second.

Response: We thank the reviewer for this comment and appreciate their positive assessment of the introduction. We have carefully considered the suggested reorganisation; however, we have retained the current structure because we feel it provides a more logical progression from the broader theoretical context of the coevolution of sociality and intelligence to the more specific role of communication as an important social-cognitive domain through which this relationship can be tested. We believe this structure best reflects the theoretical rationale and aims of our study.

Comment 2: The X2 test results: The authors’ response regarding the low expected cell counts and how they relied on Monte Carlo simulated p-values sufficiently addresses my comment. My other concern regarding the non-independence of observations and how that violates the assumptions of the test, still stand. I appreciate that under the setup of data collection it was not possible to account for caller identity and that accordingly the analysis only serves an exploratory purpose. However, I do not agree that the current presentation of the results reflect this deviation from the test assumptions. The first half recommends the reader interpret the p-values cautiously, while the second half affirms the results to reveal “clear, non-random structuring of call use across behavioural contexts. This phrasing, along with the first two lines presenting the result assert a stronger claim than supported by the results of the X2 test, making the two statements presenting this result contradictory. The authors can still state the data descriptively show a strong distinction of call type production within context, without making an unsupported statement about statistical inference. The observed pattern of call production within context can be skewed to a higher contribution of a large number of calls belonging to one individual for example, without such pattern being detected by the test.

Response:

We thank the reviewer for this comment and agree that, despite our intention to use the χ² analysis descriptively, the potential non-independence of observations limits the validity of its statistical inference. We have therefore removed the χ² test and associated p-value from the manuscript and now present the relationship between call types and behavioural contexts descriptively (based on the distribution show in Figure 3 and Table S6).

We have removed text relating to this statistical test in the Methods L403 and the Results L442.

Attachment Submitted filename: Response to Reviewers_PlosOne_22.09.26.docx

# Editor report: Decision Letter 2

**DOI:** 10.1371/journal.pone.0360246.r005

**Authors:** Brenton Cooper

25 Sep 2026

The complex, graded vocal repertoire of the social Rüppell’s vulture

PONE-D-26-13338R2

Dear Dr. Proudfoot,

We’re pleased to inform you that your manuscript has been judged scientifically suitable for publication and will be formally accepted for publication once it meets all outstanding technical requirements.  Thank you for your exceptional contribution to our journal.

Within one week, you’ll receive an e-mail detailing the required amendments. When these have been addressed, you’ll receive a formal acceptance letter and your manuscript will be scheduled for publication.

An invoice will be generated when your article is formally accepted. Please note, if your institution has a publishing partnership with PLOS and your article meets the relevant criteria, all or part of your publication costs will be covered. Please make sure your user information is up-to-date by logging into Editorial Manager at Editorial Manager® and clicking the ‘Update My Information' link at the top of the page. For questions related to billing, please contact billing support.

If your institution or institutions have a press office, please notify them about your upcoming paper to help maximize its impact. If they’ll be preparing press materials, please inform our press team as soon as possible -- no later than 48 hours after receiving the formal acceptance. Your manuscript will remain under strict press embargo until 2 pm Eastern Time on the date of publication. For more information, please contact onepress@plos.org.

Kind regards,

Brenton G. Cooper, Ph.D.

Academic Editor

PLOS One

Additional Editor Comments (optional):

Reviewers' comments:

# Editor report: Acceptance letter

**DOI:** 10.1371/journal.pone.0360246.r006

**Authors:** Brenton Cooper

PONE-D-26-13338R2

PLOS One

Dear Dr. Proudfoot,

I'm pleased to inform you that your manuscript has been deemed suitable for publication in PLOS One. Congratulations! Your manuscript is now being handed over to our production team.

At this stage, our production department will prepare your paper for publication. This includes ensuring the following:

* All references, tables, and figures are properly cited

* All relevant supporting information is included in the manuscript submission,

* There are no issues that prevent the paper from being properly typeset

You will receive further instructions from the production team, including instructions on how to review your proof when it is ready. Please keep in mind that we are working through a large volume of accepted articles, so please give us a few days to review your paper and let you know the next and final steps.

Lastly, if your institution or institutions have a press office, please let them know about your upcoming paper now to help maximize its impact. If they'll be preparing press materials, please inform our press team within the next 48 hours. Your manuscript will remain under strict press embargo until 2 pm Eastern Time on the date of publication. For more information, please contact onepress@plos.org.

You will receive an invoice from PLOS for your publication fee after your manuscript has reached the completed accept phase. If you receive an email requesting payment before acceptance or for any other service, this may be a phishing scheme. Learn how to identify phishing emails and protect your accounts at https://explore.plos.org/phishing.

If we can help with anything else, please email us at customercare@plos.org.

Thank you for submitting your work to PLOS One and supporting open access.

Kind regards,

PLOS One Editorial Office Staff

on behalf of

Dr. Brenton G. Cooper

Academic Editor

PLOS One
